# A pan-cancer bioinformatic analysis of the carcinogenic role of SMARCA1 in human carcinomas

**DOI:** 10.1371/journal.pone.0274823

**Published:** 2022-09-20

**Authors:** Lei Dai, Joseph Mugaanyi, Tongyue Zhang, Jingshu Tong, Xingchen Cai, Caide Lu, Changjiang Lu

**Affiliations:** 1 Department of Hepatopancreatobiliary Surgery, Ningbo Medical Centre Lihuili Hospital, Ningbo University, Ningbo, Zhejiang, China; 2 Tongji Hospital of Tongji Medical College, Huazhong University of Science and Technology, Wuhan, Hubei, China; King Faisal Specialist Hospital and Research Center, SAUDI ARABIA

## Abstract

SMARCA1is a mammalian imitation switch (ISWI) gene that encodes for SNF2L. SNF2L is involved in regulating cell transition from a committed progenitor state to a differentiated state. Although many papers have detailed the correlation between SMARCA1 and different cancers, no pan-cancer analysis has been conducted to date. We started by exploring the potential carcinogenic role of SMARCA1 across 33 carcinomas using the cancer genome atlas (TCGA) and the genotype-tissue expression (GTEx) databases. The expression of SMARCA1 was significantly elevated in some tumor types but not in others. There was a distinct relationship between SMARCA1 expression and patient prognosis. S116 phosphorylation levels were up-regulated in both lung adenocarcinoma and uterine corpus endometrial carcinoma. The expression level of SMARCA1 was positively correlated with cancer-associated fibroblasts infiltration in a number of tumors, such as colon adenocarcinoma, cervical squamous cell carcinoma and endocervical adenocarcinoma. It was also associated with CD8+ T-cell infiltration in head and neck squamous cell carcinoma and lung adenocarcinoma. Furthermore, SMARCA1 is involved in chromatin remodeling and protein processing-associated mechanisms. Our study presents an initial assessment and illustration of the carcinogenic role of SMARCA1 in different carcinomas.

## 1. Introduction

Oncogenesis and tumor progression mechanisms are very complex and involve many different genes. It is therefore essential to perform pan-cancer analysis of the involved genes to investigate any potential relationship between the mechanisms and patient prognosis. The cancer genome atlas (TCGA) project, the genotype-tissue expression (GTEx) and the Oncomine databases are three publicly accessible databases that provide an extensive collection of tumor-related functional genomics datasets [1–5] for researchers to carry out a pan-cancer analysis.

SWI/SNF-related, matrix-associated, actin-dependent regulator of chromatin, subfamily A, member 1 (SMARCA1) protein is an important subunit of the SWI/SNF complex involved in chromatin remodeling and transcriptional activation [6, 7]. The structure and function of SMARCA1 have been partially explored from an epigenetic and clinicopathological perspective [6–11]. Although the SMARCA1 protein has only been partially mapped, five conserved domains have been identified which are: DNA-binding domain (DBINO), HAND domain, SLIDE domain, Helicase superfamily c-terminal domain (HELICc) as well as the ’SWI3, ADA2, N-CoR and TFIIIB’ DNA-binding domains (SANT) [12–14]. Besides its correlation with non-neoplastic diseases [7, 10, 11, 15], SMARCA1 has also been observed in numerous malignancies, such as soft tissue sarcoma [16], colorectal cancer [17], gastric cancer [18], and urothelial cancer [19]. However, a comprehensive big data-based pan-cancer analysis of the correlation between SMARCA1 and different types of carcinomas is yet to be undertaken. This study outlines the pathways mediated by SMARCA1 in different disorders and cancers based on present cell- or animal-experimental evidence (S1 Fig).

We conducted a pan-cancer analysis of SMARCA1 based on the TCGA and GTEx databases and investigated the underlying molecular mechanisms of SMARCA1 in carcinogenesis, clinical prognosis and targeted therapy of various carcinomas.

## 2. Materials and methods

The methods are summarized in S2 Fig.

### 2.1 Genome and proteomic structure analysis

Genome annotations of the SMARCA1 gene were obtained from the University of California Santa Cruz (UCSC) genome browser (http://genome.ucsc.edu/) according to the human Dec 2013 (GRch38/hg28) assembly [20]. Analysis of the conserved domain of SMARCA1 was performed using the HemoloGene database (http://www.ncbi.nlm.nih.gov/homologene/) [21]. In addition, we acquired the SMARCA1 phylogenetic tree for different species based on NCBI’s constraint-based multiple alignment tool (COBALT) which computed protein sequence alignment using conserved domains and local sequence similarity information (http://www.ncbi.nlm.nih.gov/tools/cobalt) [22].

### 2.2 Gene expression analysis with HPA

The human protein atlas (HPA) database [23], a spatial map of the human proteome (http://www.proteinatlas.org/humanproteome/pathology) was used to ascertain the physiological and pathological expression data of the SMARCA1 gene in different tissues and cells by inputting the term “SMARCA1”. “Low specificity” was considered as “Normalized expression (NX) ≥ 1 in at least one tissue/region/cell type without being elevated. “Enhanced” was when “NX levels of a group (of 1–5 tissues or 1–10 cell types or 1–5 brain regions) elevated to at least four times the mean of other tissue/region/cell types”. Detailed information at: https://www.proteinatlas.org/ENSG00000102038-SMARCA1.

### 2.3 Gene expression analysis with TIMER2

The tumor immune estimation resource (TIMER), version 2.0 database (http://timer.comp-genomics.org), based on TCGA, is a comprehensive web resource for the systematical evaluation of the clinical relevance of tumor-immune infiltrates and differential gene correlation analysis [24]. We used TIMER 2.0’s *“Gene_DE” module* to explore the difference in SMARCA1 expression between tumor and adjacent normal tissues.

### 2.4 Gene expression analysis with GEPIA2

Gene expression profiling interactive analysis (GEPIA), version 2.0 is a public (http://gepia.cancer-pku.cn/index.html) web application based on GTEx and TCGA databases [25]. We used it to assess further the difference in expression of SMARCA1 between tumor and the corresponding normal tissues, for tumors with significantly restricted normal or without normal tissues in TIMER2.0 [e.g., Adrenocortical Carcinoma (ACC), Lower Grade Glioma (LGG), etc.]. Configuration was as follows: the cutoff of Log_2_FC (fold change) and *P*-value were 1 and 0.01 respectively, and TCGA normal and GTEx data were matched. Additionally, we evaluated the SMARCA1 expression level in each pathological stage of all TCGA carcinomas using the “Expression DIY-Stage plot” module of GEPIA2. The log_2_(TPM (Transcripts per million) + 1) for the log-scale was applied in assessments.

### 2.5 Gene expression analysis with UALCAN

UALCAN (http://ualcan.path.uab.edu/index.html) is a comprehensive and interactive public resource for cancer OMICS data analysis [26], provided by the Clinical proteomic tumor analysis consortium (CPTAC) dataset [27]. We used it to conduct translational level analysis of the SMARCA1 gene. Using “SMARCA1” as the search keyword, we explored and compared the total protein expression levels (NP_001269803.1) between normal and primary tumor tissues of the five available tumors (breast cancer, ovarian cancer, clear cell renal cell carcinoma (RCC), uterine corpus endometrial carcinoma (UCEC) and lung adenocarcinoma (LUAD)).

### 2.6 Gene expression analysis with Oncomine

For further verification, the Oncomine database, currently the world’s largest database of oncogene chips and features an integrated data mining platform (https://www.oncomine.org) [5], was used to assess the difference in SMARCA1 expression between tumor and normal tissues under the default settings (with a threshold of “FC = 1.5, *P*-value = 0.05, and Gene rank = Top 10%”). Several comparisons across at least 6 analyses were obtained with the included studies’ median rank, P-value, and legends.

### 2.7 Prognosis—survival analysis

Correlation maps for SMARCA1 expression, overall survival (OS) and disease-free survival (DFS) for cancers in TCGA were obtained using the “Survival Map” module of GEPIA2 [25]. The median, estimated by the Mantel-Cox test was used as the cutoff value (cutoff-high = 50%, cutoff-low = 50%). Subsequently, relevant Kaplan-Meier curves were obtained using the same module with the log-rank test and default parameters.

The Kaplan-Meier Plotter database [28] (http://kmplot.com/analysis) was constructed based on gene microarray and RNA-seq data from the gene expression omnibus (GEO) [29], European genome-phenome archive (EGA) [30], and TCGA public databases. We conducted a series of survival analyses of the relationships between SMARCA1 and various cancers to determine OS, relapse-free survival (RFS), distant metastasis-free survival (DMFS), post-progression survival (PPS), progress-free survival (PFS), first progression (FP) and disease-specific survival (DSS). The parameters were as follows: Affymetrix id/Gene symbol: use mean expression of selected genes (203873_at, 203874_s_at, 203875_at, 215294_s_at) in breast, ovarian, lung and gastric cancers; use RNA-seq id_6594 in liver cancer; patients split by auto-select best cutoff; HR: yes; 95% confidence interval (CI): yes; with the remaining settings set to their default values.

We also screened the clinical indicators for the subgroup analyses to find out the high-risk factors related to the SMARCA1 expression and prognosis of different cancers [e.g., stage, grade, AJCC_T, gender, race, alcohol consumption, intrinsic subtype, surgery, or chemotherapy, etc.]. Then we imported the metan module of STATA 15.0 software (College Station, TX, StataCorp LP, USA) [31] to perform a meta-analysis of the above data. Z-test was used and a forest plot was plotted.

### 2.8 Genetic alteration analysis

Using cBioPortal (http://www.cbioportal.org) for cancer genomics visualization [32–34], we selected “Quick select; TCGA PanCancer Atlas Studies” on the home page and queried for “SMARCA1” genetic variation characteristics. Data including alteration frequency, mutation type and copy number alteration (CNA) for cancers in TCGA was extracted. Next, mutated site results of SMARCA1 exhibited in the pattern chart of the three-dimensional (3D) or protein structure through the “Mutations” module were obtained. Differences in OS, DFS, PFS and DSS with and without SMARCA1 gene variation were compared using the “Comparison” module.

### 2.9 DNA methylation analysis

We conducted DNA methylation analysis for the SMARCA1 gene for numerous probes (e.g., cg10076009, cg18751555, etc.) and different cancers using the MEXPRESS database (http://mexpress.be/) [35]. In MEXPRESS, DNA methylation, expression and clinical data was visualized, as well as Pearson correlation coefficient R-values and Benjamini-Hochberg-adjusted P-values were analyzed.

### 2.10 Phosphorylation feature analysis

We also explored the expression level of SMARCA1 of the phospho-protein (at the S116 site) using UALCAN. We retrieved the predicted phosphorylation features of the site via querying for “SMARCA1” in the PhosphoNET database (http://www.phosphonet.ca/), an open-access human phosphosite knowledgebase [36].

### 2.11 Tumor-related immune infiltration analysis

We selected cancer-associated fibroblasts and the immune cells of CD8^+^ T-cells in the “immune-Gene” module of the TIMER2 database to assess the potential correlation between SMARCA1 expression and tumor-related immune infiltrates across 33 cancers in TCGA. We visualized the data with heatmaps [24] and scatter plots via immune infiltration evaluation with the QUANTISEQ, TIMER, MCPCOUNTER, XCELL, CIBERSORT, CIBERSORT-ABS, TIDE and EPIC algorithms applied by purity-adjusted Spearman’s rank correlation test.

### 2.12 Gene-immune analysis

Sangerbox database (http://www.sangerbox.com), based on TCGA and GEO databases, is a practical bioinformation analysis tool [37]. We used it to analyze the potential relationship between SMARCA1 expression and microsatellite instability (MSI) or tumor mutational burden (TMB). The partial correlation value (cor) and ***P***-value were calculated using Spearman’s rank test and circle plots.

### 2.13 Gene enrichment analysis

The STRING database (http://string-db.org/) [38], which supports the functional proteins association networks, was used for SMARCA1-related proteomic analysis. We set the main parameters as follows: protein name (“SMARCA1”), organism (“Homo sapiens”), the meaning of network edges (“evidence”), active interaction sources (“Experiments”), the minimum required interaction score [“low confidence (0.150)”] and max number of interactors to show [“1^st^ shell: no more than 50 interactors”]. Finally, we acquired the SMARCA1-related protein networks diagram by enrichment analysis.

First, we retrieved data for the top 100 SMARCA1-related/similar genes via the “similar genes detection” module of GEPIA2 based on all tumor and normal tissues in TCGA. Then we performed a pairwise Pearson correlation analysis of SMARCA1 and the selected genes using the “correlation analysis” module of GEPIA2. Afterward, we applied the “Gene_corr” module of TIMER2 to perform the purity-adjusted Spearman’s rank correlation analysis and the selected genes in a heatmap with statistically significant data. This was followed by interaction analysis of SMARCA1-correlated and interacted genes on Jvenn (http://jvenn.toulouse.inra.fr/app/example.html), which is an interactive Venn diagram viewer [39]. We combined the two gene lists to conduct a Kyoto encyclopedia of genes and genomes (KEGG) pathway analysis visualized with the “tidyr” and “ggplot2” packages in R. The data was obtained from the functional annotation chart using the DAVID Bioinformatics Resources 6.8 [40, 41] by uploading gene lists, followed by its analysis wizard. We also conducted Gene ontology (GO) enrichment analysis using R’s (version 4.0.3, www.r-project.org) [42] “clusterProfiler” package. All the data analysis output visualized cnetplots (node_label = T, colorEdge = T, circular = F), including biological process (BP), cellular component (CC) and molecular function (MF). Two-tailed *P* value less than 0.05 is regarded as statistically significant.

### 2.14 Diagnostic capability analysis of SMARCA1

Based on the TCGA and GTEx database data, we conducted a diagnostic capability analysis of SMARCA1 pan-cancer using receiver operating characteristic (ROC) curves. The area under curve (AUC) was calculated for evaluating the diagnostic value of SMARCA1.

### 2.15 Statistical analysis

All statistical analyses were performed with R (version 4.0.3). Normally distributed variables were analyzed using the t-test and one-way ANOVA test and non-normally distributed variables with nonparametric tests. Log-rank test and cox regression were used for survival analysis, Pearson’s correlation and spearman’s rank correlation test for correlation analysis. p-value < 0.05 was considered statistically significant.

## 3. Results

### 3.1 Gene expression analysis

This study aimed to assess the tumorigenic role of human SMARCA1 (NM_003069.5 for mRNA or NP_003060.2 for protein, S3A Fig). The conserved domains of SMARCA1 protein structure are similar among different species (e.g., X.tropicalis, D.rerio, H.sapiens, etc.), such as HAND (pfam09110), HELICc (cd00079) and SANT (cl17250) domains (S3B Fig). The phylogenetic tree (S4 Fig) demonstrated an evolutionary correlation of SMARCA1 in different species.

**Fig 1:**
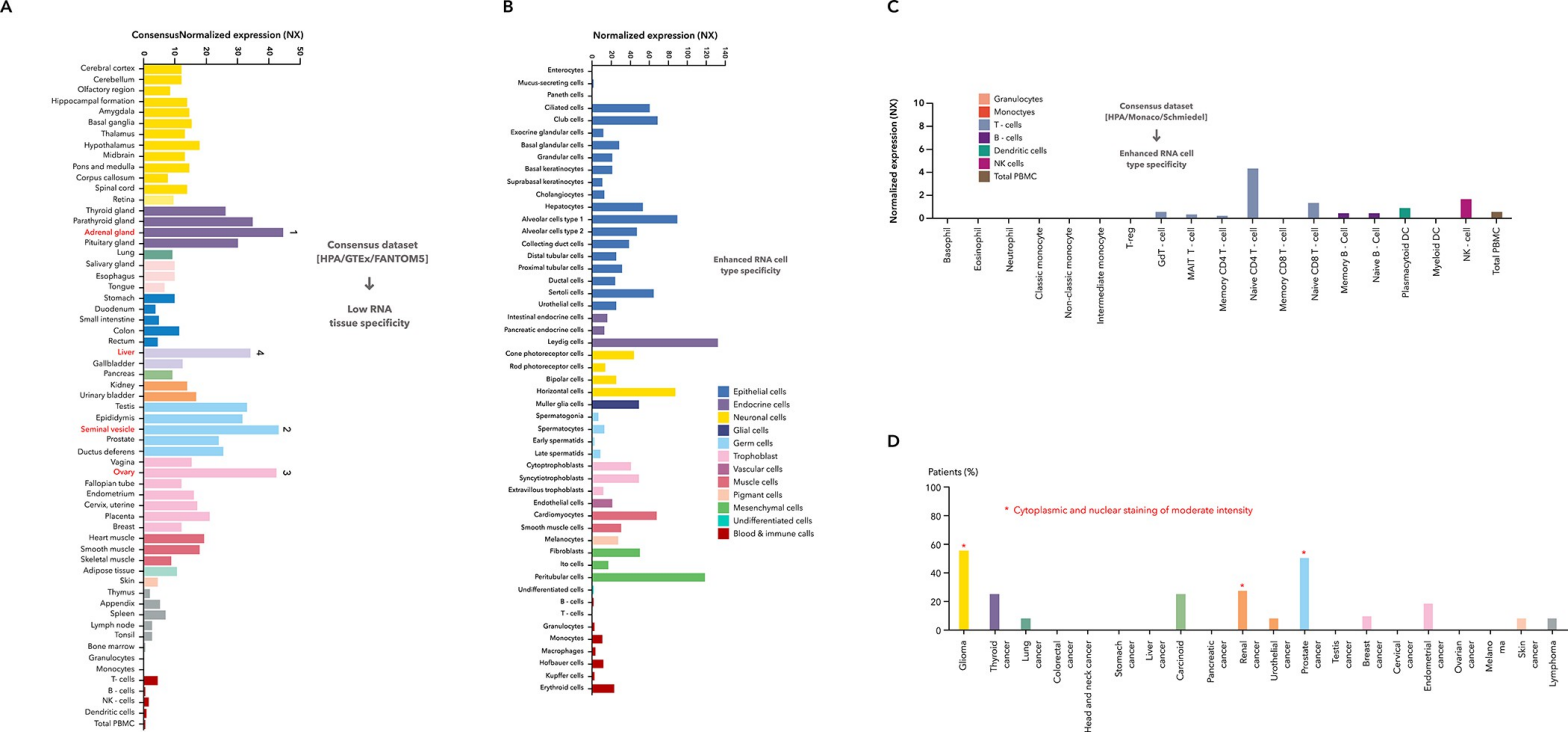
SMARCA1 expression in different cells, tissues in the physiological and pathological status. (A) Analysis of SMARCA1 gene expression in different tissues by the consensus datasets of HPA, GTEx and FANTOM5; (B) Analysis of SMARCA1 gene expression in different single cell types; (C) Analysis of SMARCA1 gene expression in different blood cells by the consensus datasets of HPA, Monaco and Schmiedel. (D) Analysis of SMARCA1 gene expression in different cancer tissues.

We obtained the RNA expression pattern of SMARCA1 in different single cell types and normal tissues based on the combined analysis of the GTEx, Human protein atlas (HPA) and Function annotation of the mammalian genome 5 (FANTOM5) datasets. SMARCA1 expression is highest in the adrenal gland, followed by the seminal vesicle, ovary and liver (Fig 1A) by tissue and in Leydig and peritubular cells by cell type (Fig 1B). Although SMARCA1 expression can be observed in nearly all the tissues detected (consensus normalized expression (NX) > 1), low RNA tissue specificity and enhanced RNA cell type specificity appears both in different single cell types and blood cells (Fig 1C). Furthermore, SMARCA1 expression in the cytoplasm and nucleus reaches moderate intensity in glioma, prostate and renal (Fig 1D).

**Fig 2:**
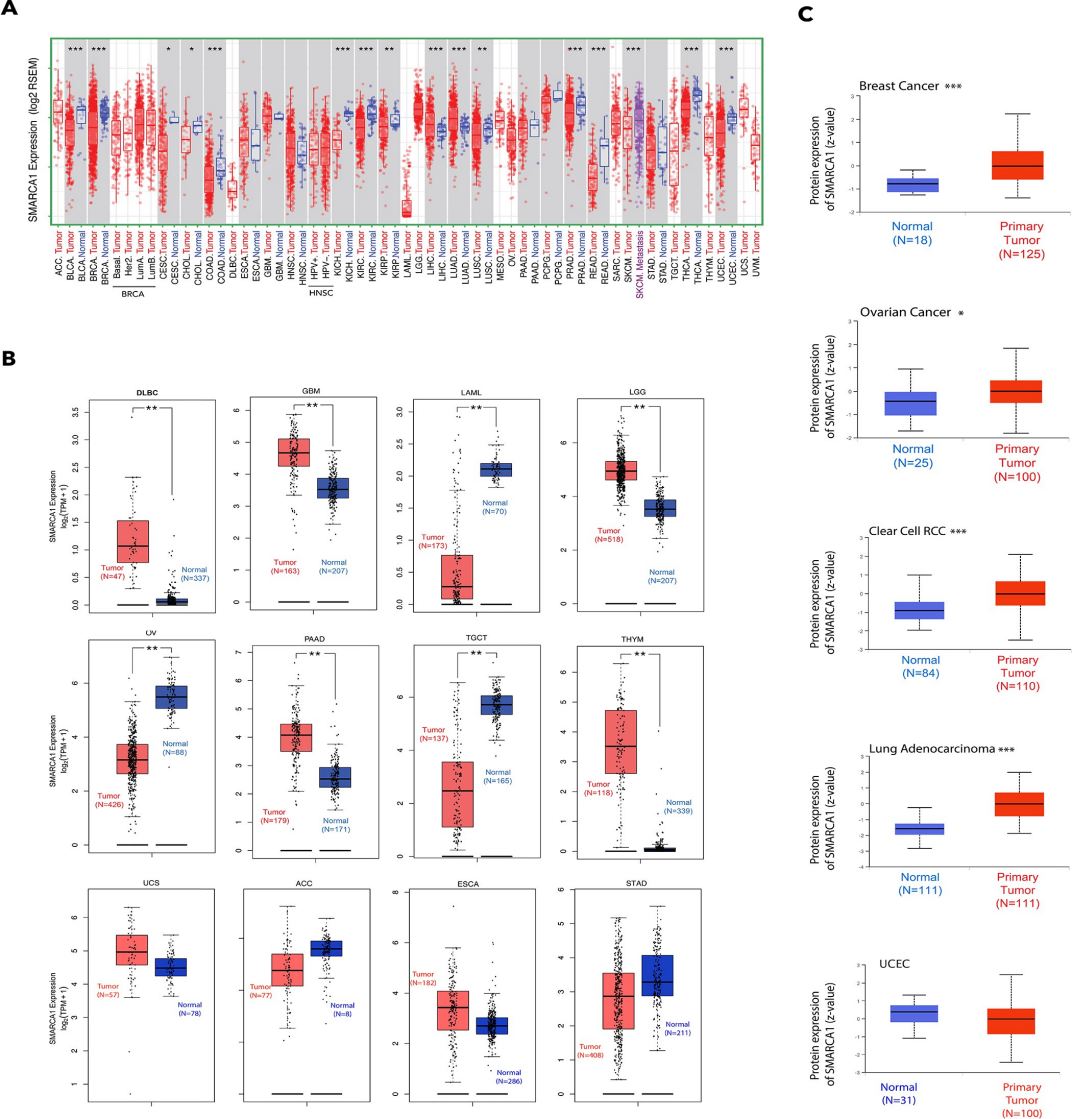
Comparison of SMARCA1 expression level in different tumor and adjacent normal tissues. (A) Analysis of SMARCA1 expression level in different tumors or their subtypes and corresponding normal tissues via TIMER2. * *P* < 0.05, ** *P* < 0.01, *** *P* < 0.001. (B) By including the relevant normal tissues of the GTEx database as controls, the SMARCA1 Expression status in ACC, DLBC, GBM, ESCA, LAML, LGG, OV, PAAD, STAD, TGCT, THYM and UCS were analyzed via GEPIA2. ** *P* < 0.01. (C) Analysis of SMARCA1 expression in total protein between primary tumor and normal tissues of five cancers based on the CPTAC dataset. * *P* < 0.05, ** *P* < 0.01, *** *P* < 0.001.

We analyzed SMARCA1 expression levels in all tumors of TCGA via TIMER2. As shown in Fig 2A, the level of SMARCA1 expression in the cancer tissues of bladder urothelial carcinoma (BLCA), breast invasive carcinoma (BRCA), colon adenocarcinoma (COAD), kidney chromophobe (KICH), kidney renal clear cell carcinoma (KIRC), prostate adenocarcinoma (PRAD), rectum adenocarcinoma (READ), skin cutaneous carcinoma (SKCM), thyroid carcinoma (THCA), uterine corpus endometrial carcinoma (UCEC) (*P* < 0.001), kidney renal papillary cell carcinoma (KIRP), lung squamous cell carcinoma (LUSC) (*P* < 0.01), cervical squamous cell carcinoma and endocervical adenocarcinoma (CESC) and cholangiocarcinoma (CHOL) (*P* < 0.05) is lower than the adjacent normal tissues. On the contrary, the expression in the tumor tissues of lung adenocarcinoma (LUAD) and liver hepatocellular carcinoma (LIHC) is higher.

We proceeded to include data of the normal tissues from the GTEx database as controls for evaluating the difference in SMARCA1 expression between tumor and normal tissues of diffuse large B cell lymphoma (DLBC), glioblastoma multiforme (GBM), acute myeloid leukemia (LAML), brain lower grade glioma (LGG), ovarian serous cystadenocarcinoma (OV), pancreatic adenocarcinoma (PAAD), testicular germ cell tumors (TGCT), and thymoma (THYM) (*P* < 0.01). However, we did not observe a significant difference in other cancers, including adrenocortical carcinoma (ACC), esophageal carcinoma (ESCA), stomach adenocarcinoma (STAD) and uterine carcinosarcoma (UCS) (Fig 2B).

**Fig 3:**
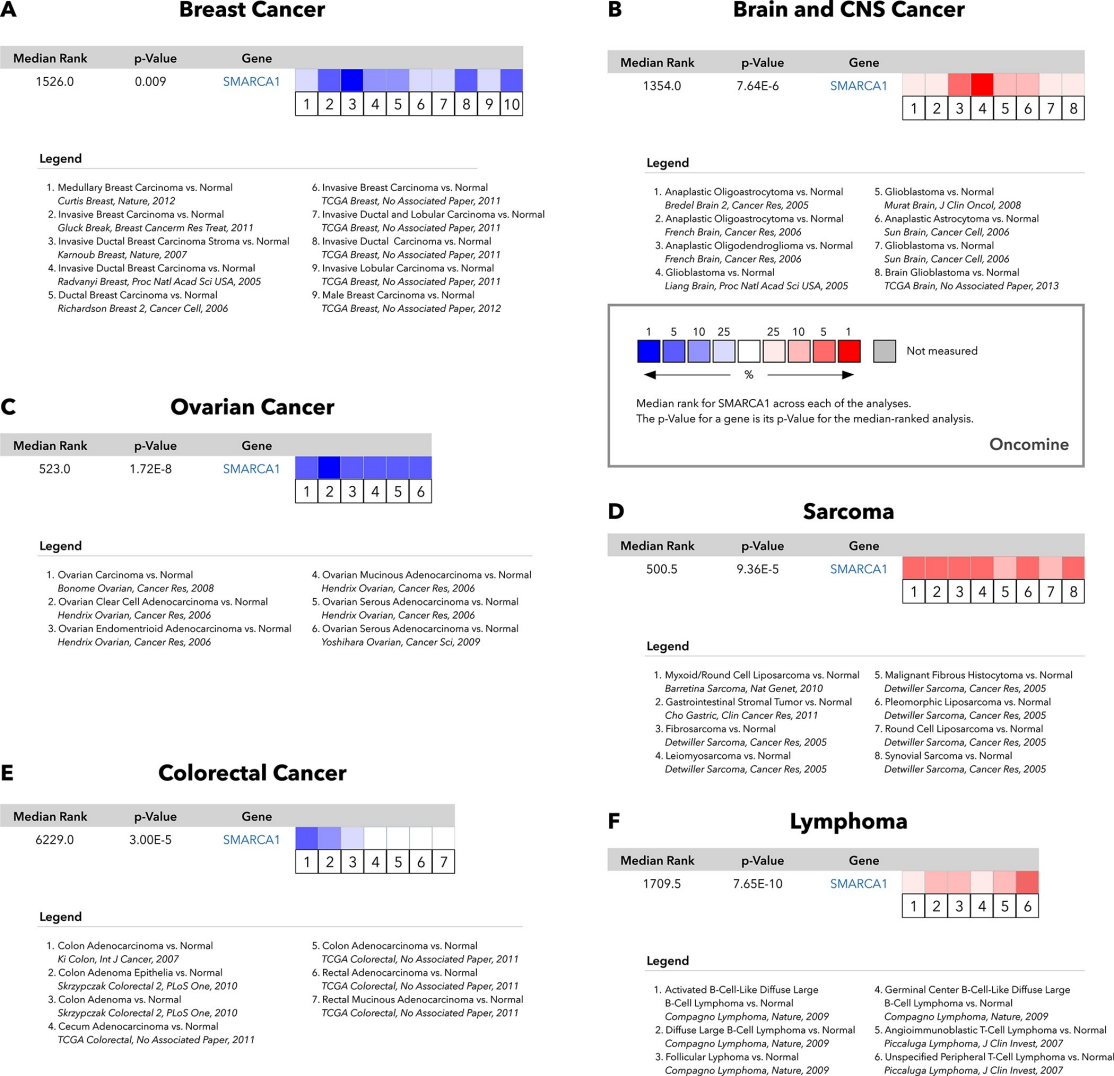
Pooling analysis of the difference in SMARCA1 expression between tumor and normal tissues via the Oncomine database. (A) Breast cancer; (B) Brain and CNS cancer; (C) Ovarian cancer; (D) Sarcoma; (E) Colorectal cancer; (F) Lymphoma.

According to the analysis using the CPTAC database, we found that SMARCA1 total protein expression was higher in the primary tumor tissues of breast cancer, clear cell RCC, LUAD (*P* < 0.001), and ovarian cancer (*P* < 0.05) than in normal tissues except in the case of UCEC (Fig 2C). The results of pooling analysis in the Oncomine database further confirmed the findings for breast cancer, ovarian cancer, and colorectal cancer (all *P* < 0.01). However higher expression was observed in brain and CNS cancer, sarcoma and lymphoma (all *P* < 0.001) compared to the control (Fig 3).

**Fig 4:**
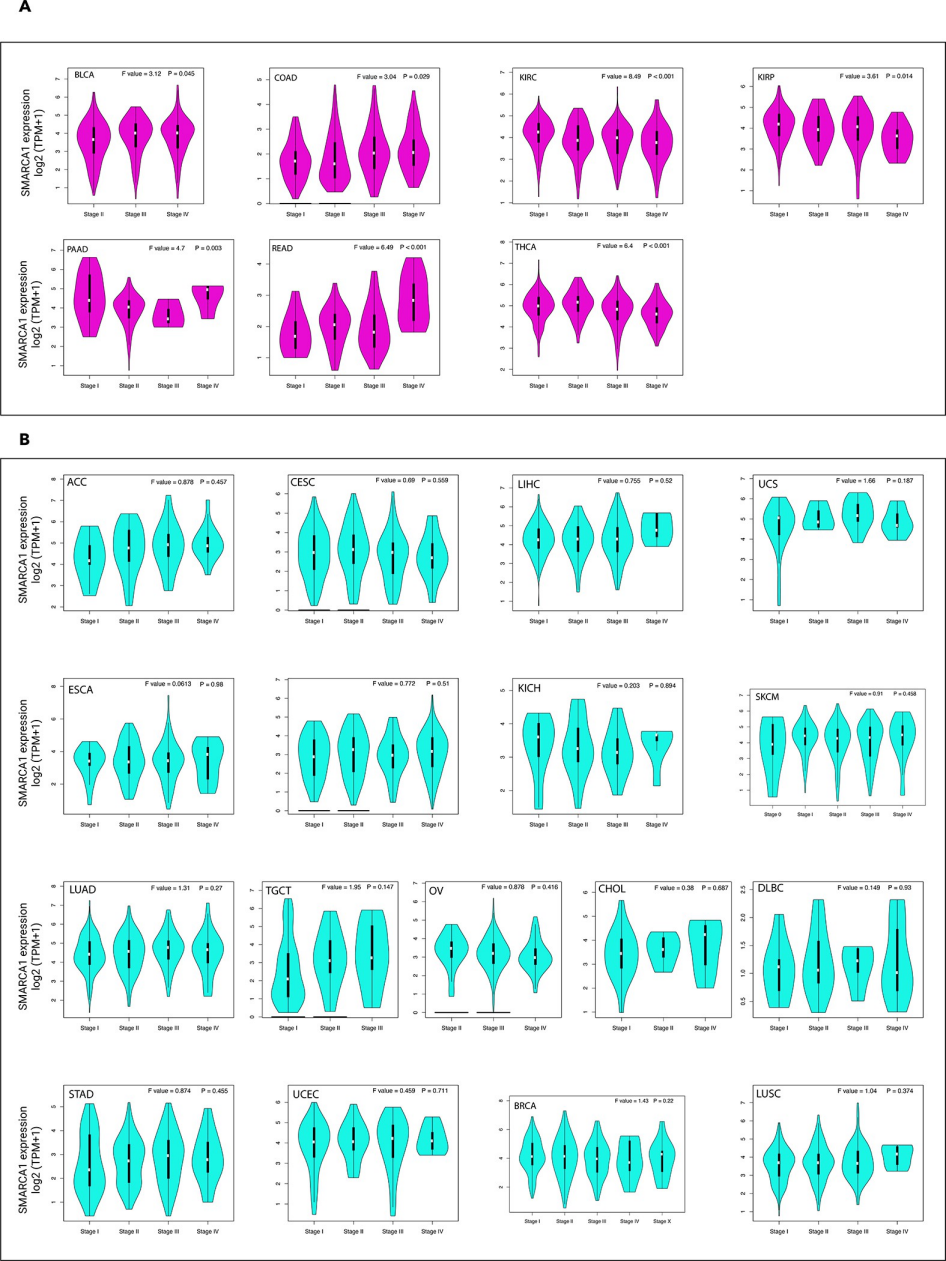
Expression of SMARCA1 gene in different pathological stages of diverse cancers in TCGA. (A) Expression difference in BLCA, COAD, KIRC, KIRP, PAAD, READ and THCA; (B) Expression difference in ACC, BRCA, CESC, CHOL, DLBC, ESCA, HNSC, KICH, LIHC, LUAD, LUSC, OV, SKCM, STAD, TGCT, UCEC and UCS. Log_2_(TPM+1) was applied for log-scale, * *P* < 0.05, ** *P* < 0.01, *** *P* < 0.001.

We also investigated the relationship between SMARCA1 expression and different pathological stages of various cancers via the “pathological stage plot” module of GEPIA2. Significant differences were observed in BLCA, COAD, KIRP, KIRC, PAAD, READ and THCA (all *P* < 0.05) (Fig 4A) but not in others (Fig 4B).

### 3.2 Survival analysis

**Fig 5:**
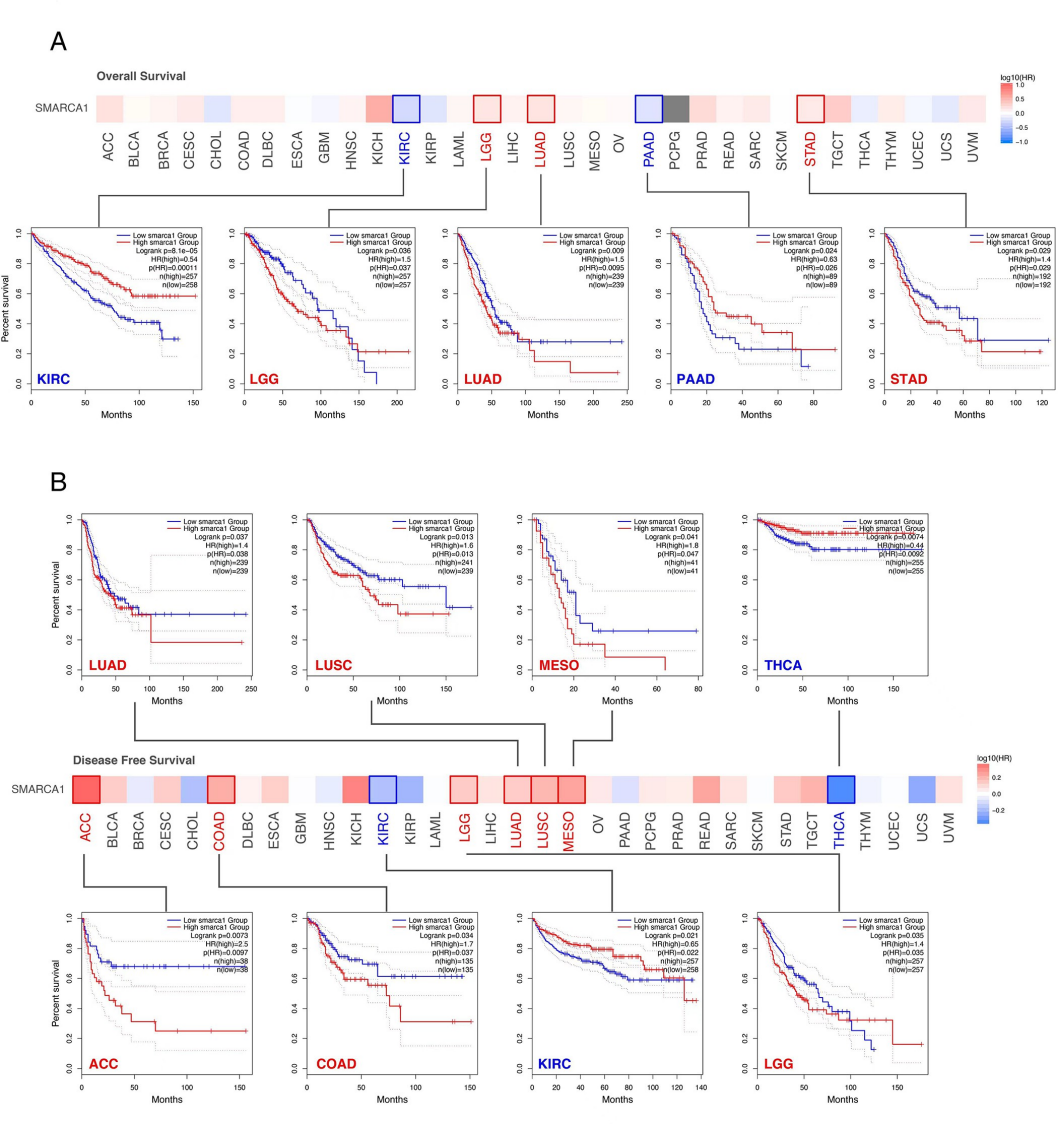
Analysis of SMARCA1 expression associated prognosis different cancers via GEPIA2. (A) Overall survival (OS) analyses between SMARCA1 expression and all cancers included; (B) disease-free survival (DFS) analyses between SMARCA1 expression and all cancers included with demonstration of survival map and Kaplan-Meier curves.

We divided the cases into two groups according to high or low expression of SMARCA1 to assess the relationships between SMARCA1 expression and patient prognosis in different cancers, based on the TCGA and GEO databases. High expression of SMARCA1 was related to poor overall survival (OS) in patients with LGG (*P* = 0.036), LUAD (*P* = 0.009), and STAD (*P* = 0.029). Low expression was associated with poor OS in KIRC (*P* < 0.001) and PAAD (*P* = 0.024) (Fig 5A). Similarly, high expression of SMARCA1 was associated with poor disease-free survival (DFS) for LUAD (*P* = 0.037),lung squamous cell carcinoma (LUSC) (*P* = 0.013), mesothelioma (MESO) (*P* = 0.041), adrenocortical carcinoma (ACC) (*P* = 0.0073), COAD (*P* = 0.034) and LGG (*P* = 0.035), low expression in THCA (*P* = 0.0074) and KIRC (*P* = 0.021) (Fig 5B).

**Fig 6:**
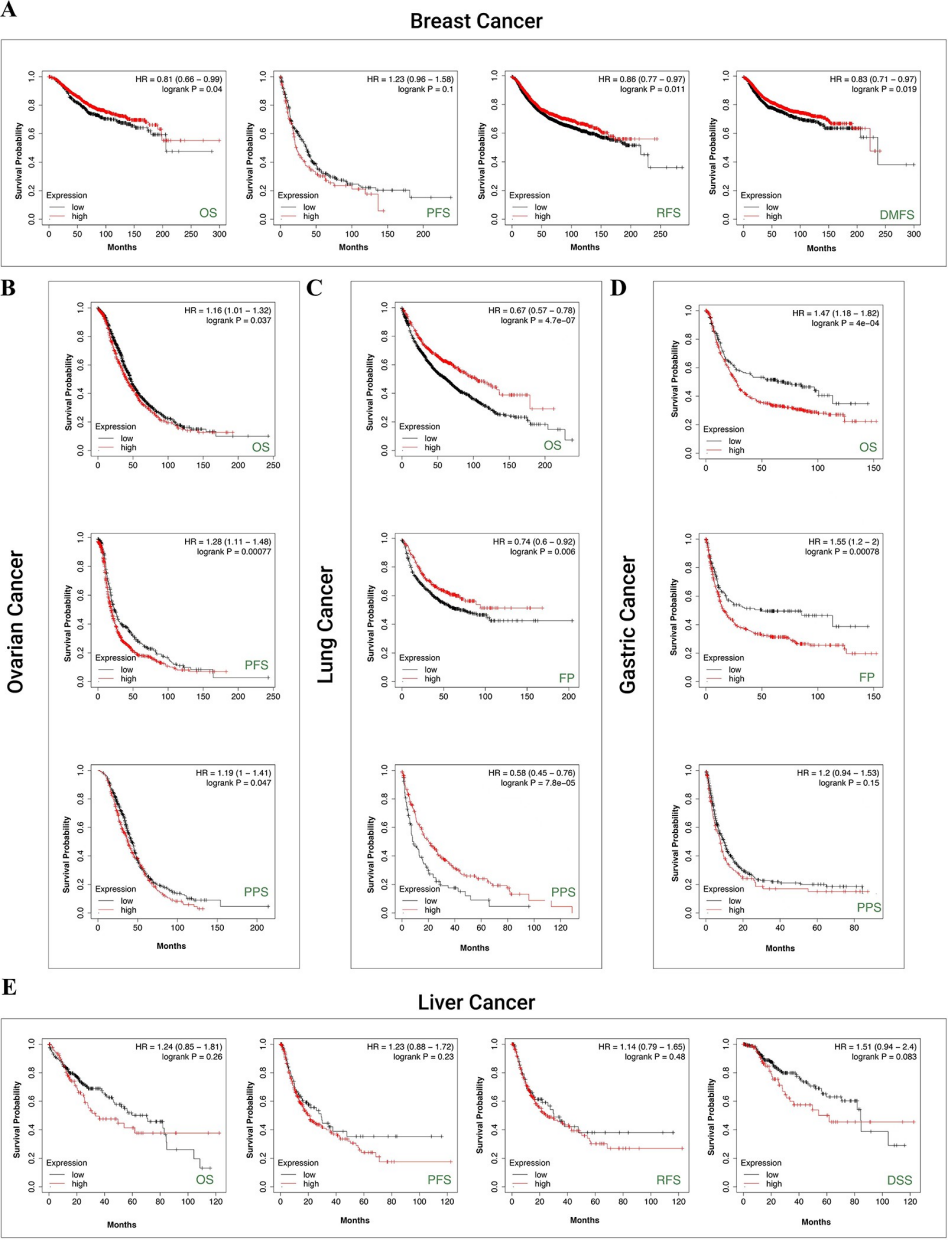
Correlation analysis between SMARCA1 expression and prognosis of different cancers via Kaplan-Meier plotter. (A) OS, PPS, RFS, DMFS in breast cancer; (B) OS, PFS, PPS in ovarian cancer; (C) OS, FP, PPS in lung cancer; (D) OS, FP, PPS in gastric cancer; (E) OS, PFS, RFS, DSS in liver cancer. OS, overall survival; PPS, post progression survival; RFS, relapse free survival; DMFS, distant metastasis free survival; FP, first progression; PFS, progress free survival; DSS, disease-specific survival.

We used the Kaplan-Meier plotter to evaluate the correlation between SMARCA1 expression and five cancers. For breast cancer, low SMARCA1 expression was linked to poor OS (*P* = 0.04), relapse-free survival (RFS) (*P* = 0.011) and distant metastasis-free survival (DMFS) (*P* = 0.019) but not progress-free survival (PFS) (Fig 6A). The same was observed in OS (*P* < 0.001), first progression (FP) (*P* = 0.006) and post-progression survival (PPS) (*P* < 0.001) of lung cancer (Fig 6C). In contrast, high SMARCA1 expression was related to poor OS (*P* = 0.037), PFS (*P* < 0.001) and PPS (*P* = 0.047) in ovarian cancer (Fig 6B), as well as to OS (*P* < 0.001), FP (*P* < 0.001) in gastric cancer (Fig 6D). No significant difference based on SMARCA1 expression in relation to OS, PFS, RFS and disease-specific survival (DSS) was observed in liver cancer (all *P* > 0.05) (Fig 6E).

**Fig 7:**
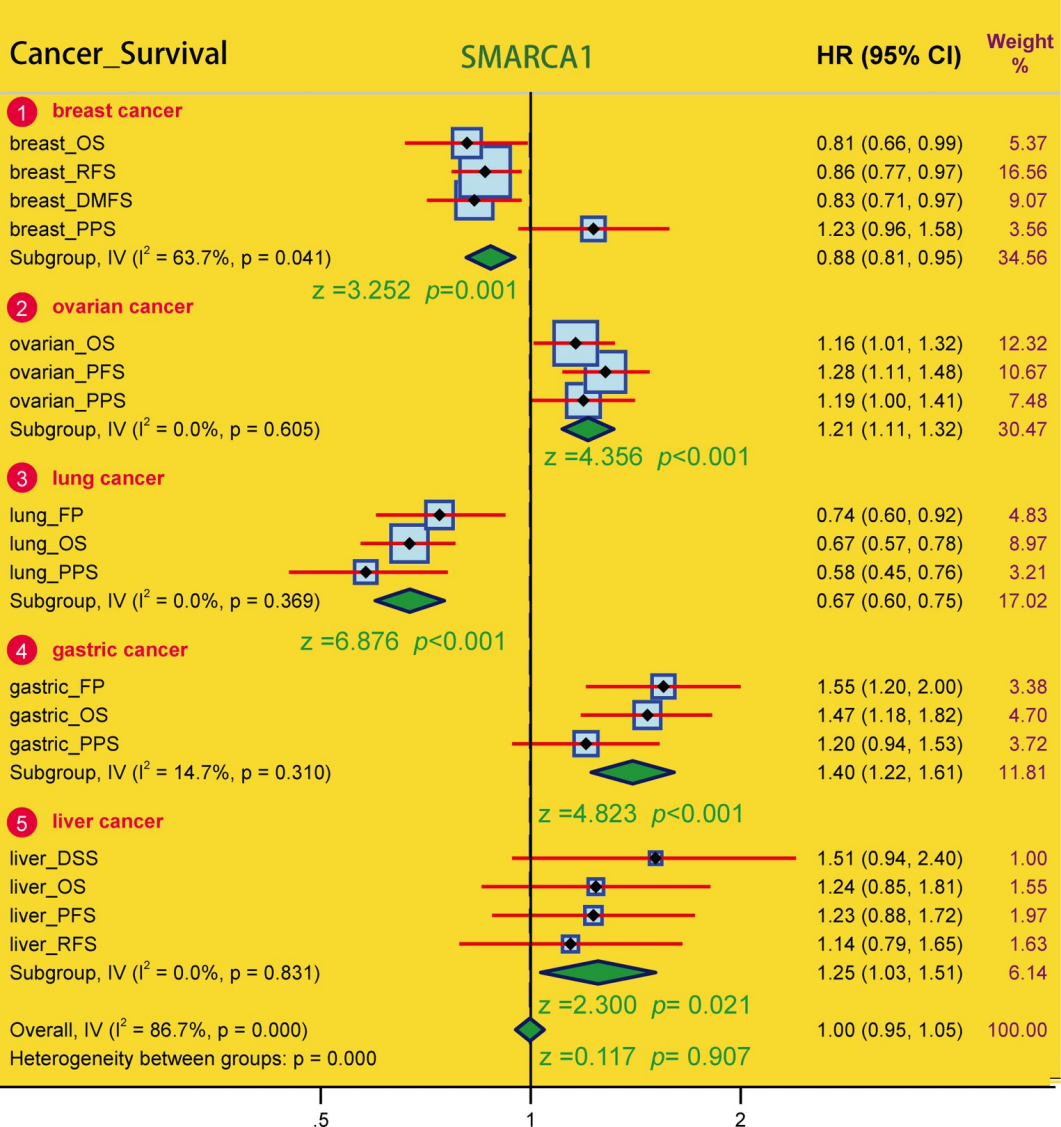
Meta-analysis on the relationship between SMARCA1 expression and multiple factors of various tumor prognosis. A series of subgroups survival analyses in breast, ovarian, lung, gastric and liver cancer were conducted with z-test, *P < 0.05, **P < 0.01, ***P < 0.001.

We performed a meta-analysis (Fig 7) to validate the correlation observed in breast cancer (*P* = 0.001), lung cancer, ovarian cancer and gastric cancer (all *P* < 0.001) again and to identify any potential SMARCA1-prognosis correlation in liver cancer (*P* = 0.021). We performed a series of subgroup analyses through restricted sub-types or select cohorts and obtained distinct findings (Tables 1–5). We found SMARCA1-associated prognosis to correlate with tumor type and clinical risk factors.

**Table 1:** Subgroup analysis on the correlation of *SMARCA1* expression and prognosis of breast cancer cases.

Factor	Subgroup	Sample size	OS		RFS		PPS		DMFS	
			HR	*P*	HR	*P*	HR	*P*	HR	*P*
**ER status-IHC**	ER positive	3499	0.72	**0.038**	0.87	0.11	1.35	0.18	0.82	0.16
	ER negative	2168	1.32	0.11	1.18	0.12	0.65	0.098	1.14	0.37
**ER status-array**	ER positive	5526	1.16	0.24	0.85	**0.014**	1.38	**0.019**	0.82	0.051
	ER negative	2009	1.22	0.27	1.1	0.3	0.69	0.11	1.24	0.12
**TP53 status**	mutated	272	1.76	0.1	1.63	**0.049**	0.68	0.44	2.21	**0.033**
	Wild type	388	0.44	**0.0085**	0.81	0.31	0.48	**0.038**	0.34	**0.0022**
**PR status-IHC**	PR positive	1559	0.71	0.37	0.73	**0.032**	0.41	0.11	0.57	**0.029**
	PR negative	1989	1.46	0.13	0.83	0.13	2.95	**0.029**	0.78	0.096
**HER2 status-array**	HER2 positive	1273	0.7	0.064	0.85	0.13	0.83	0.43	0.99	0.95
	HER2 negative	6262	1.13	0.28	0.86	**0.026**	1.35	**0.039**	0.82	**0.026**
**Grade**	Grade 1	576	0.68	0.39	0.55	**0.02**	0.39	0.13	0.44	0.095
	Grade 2	1795	0.58	**0.0069**	0.73	**0.0048**	0.72	0.17	0.7	**0.019**
	Grade 3	2058	1.31	0.11	1.17	0.12	1.3	0.21	1.19	0.22
**Intrinsic subtype**	Basal	1494	1.17	0.42	1.45	**0.0034**	0.6	**0.08**	1.26	0.16
	Luminal A	3511	1.22	0.23	0.84	**0.048**	1.41	0.059	0.76	**0.044**
	Luminal B	2015	1.2	0.31	0.88	0.15	1.46	0.079	0.8	0.12
	HER2+	515	1.79	0.056	0.87	0.47	0.77	0.49	1.51	0.11
**Lymph node status**	Lymph node positive	2153	0.73	0.057	0.79	**0.012**	0.6	**0.015**	0.72	**0.013**
	Lymph node negative	2829	0.79	0.18	0.91	0.31	0.8	0.28	0.87	0.28
**Pietenpol subtype**	Basal-like 1	418	0.52	0.11	1.5	0.14	0.2	**0.01**	0.68	0.21
	Basal-like 2	165	2.06	0.14	1.43	0.28	NA	NA	2.53	0.12
	immunomodulatory	462	2.79	**0.0084**	1.54	0.12	4.58	**0.015**	2.03	**0.0099**
	Mesenchymal	382	1.63	0.15	1.49	**0.046**	0.67	0.36	1.36	0.31
	Mesenchymal stem-like	201	4.03	**0.048**	3.69	**0.021**	NA	NA	3.04	0.071
	Luminal androgen receptor	413	0.47	**0.04**	0.57	**0.012**	0.6	0.23	0.56	0.069

HR, hazard ratio; OS, overall survival; RFS, relapse free survival; DMFS, distant metastasis free survival; PPS, post progression survival; ER, Estrogen receptor; PR, Progesterone receptor; HER2, human epidermal growth factor receptor-2; TP53, Tumor Protein P53; NA, not available data; *P* value less than 0.05 is shown in bold.

**Table 2:** Subgroup analysis on the correlation of *SMARCA1* expression and prognosis of ovarian cancer cases.

Factor	Subgroup	Sample size	OS		PFS		PPS	
			HR	*p*	HR	*p*	HR	*p*
**Histology**	Endometrioid	62	0.35	0.23	0.28	**0.0045**	NA	NA
	Serous	1232	1.22	**0.021**	1.13	0.14	1.17	0.087
**Stage**	Stage 1	107	0.68	0.53	0.41	0.23	NA	NA
	Stage 2	72	0.35	0.16	0.52	0.058	3.65	0.19
	Stage 3	1079	1.29	**0.0048**	1.11	0.2	1.22	**0.035**
	Stage 4	189	0.8	0.25	1.52	**0.038**	0.73	0.22
**Grade**	Grade 1	56	0.47	0.18	0.23	**0.035**	NA	NA
	Grade 2	325	1.23	0.19	1.45	**0.025**	1.19	0.37
	Grade 3	1024	1.19	**0.04**	1.14	0.14	1.18	0.1
	Grade 4	21	2.17	0.11	NA	NA	NA	NA
**TP53 mutation**	Mutated	516	0.91	0.43	1.35	**0.026**	0.79	0.089
	Wild type	102	2.2	**0.021**	1.72	0.058	1.79	0.11
**Debulk**	optimal	802	1.13	0.28	1.24	**0.044**	0.85	0.28
	suboptimal	536	1.31	**0.0083**	1.6	**1.3e-05**	1.49	**0.0026**
**Chemotherapy**	Contains platin	1438	1.21	**0.015**	1.35	**2.6e-05**	1.2	0.06
	Contains Taxol	821	1.27	**0.015**	1.23	**0.024**	1.29	**0.017**
	Contains Taxol+platin	804	1.28	**0.015**	1.25	**0.014**	1.31	**0.012**
	Contains Avastin	50	0.31	**0.01**	0.44	**0.019**	0.29	**0.0088**
	Contains Docetaxel	108	0.57	0.057	0.63	0.085	0.66	0.19
	Contains Gemcitabine	135	0.65	**0.033**	1.23	0.31	0.61	0.063
	Contains Paclitaxel	248	0.63	0.059	1.16	0.46	0.54	**0.032**
	Contains Topotecan	119	0.66	0.062	1.39	0.1	0.73	0.13

HR, hazard ratio; OS, overall survival; PFS, progress free survival; PPS, post progression survival; TP53, Tumor Protein P53; NA, not available data; *P* value less than 0.05 is shown in bold.

**Table 3:** Subgroup analysis on the correlation of *SMARCA1* expression and prognosis of lung cancer cases.

Factor	Subgroup	Sample size	OS		FP		PPS	
			HR	*P*	HR	HR	*P*	HR
**Histology**	adenocarcinoma	865	0.53	**2.1e-06**	0.69	**0.02**	0.55	**0.016**
	squamous cell carcinoma	675	1.22	0.11	1.94	**0.012**	1.87	0.23
**gender**	female	817	0.58	**3e-04**	0.63	**0.0074**	0.62	**0.022**
	male	1387	0.72	**1.3e-04**	0.77	0.069	0.57	**0.0015**
**smoking history**	exclude those never smoked	970	0.8	0.069	1.37	**0.015**	0.59	**0.0011**
	only those never smoked	247	0.35	**1.2e-04**	0.41	**1.3e-04**	0.4	**0.009**
**stage**	stage I	652	0.51	**6.7e-07**	0.71	0.13	0.4	**0.0021**
	stage II	320	0.56	**0.013**	2.05	**0.044**	0.47	**0.02**
	stage III	70	1.28	0.39	NA	NA	NA	NA
	stage IV	4	NA	NA	NA	NA	NA	NA
**grade**	grade I	202	1.22	0.27	0.54	**0.024**	0.81	0.45
	grade II	310	1.56	**0.0064**	1.6	**0.044**	1.47	0.15
	grade III	77	1.25	0.51	1.88	0.2	0.23	**0.022**
**AJCC stage t**	t1	475	0.71	**0.02**	2.29	**0.0021**	0.56	0.097
	t2	686	1.48	**5.5e-04**	1.74	**2.9e-04**	0.67	**0.022**
	t3	99	1.31	0.33	0.56	0.23	NA	NA
	t4	48	1.74	0.091	NA	NA	NA	NA
**AJCC stage n**	n0	863	1.13	0.34	1.75	**7e-04**	0.58	**0.0077**
	n1	296	1.19	0.28	2.31	**3.7e-4**	0.51	**0.013**
	n2	113	1.22	0.37	0.49	0.051	1.91	0.15
**AJCC stage m**	m0	818	1.16	0.16	1.67	0.062	0.62	0.16
	m1	10	NA	NA	NA	NA	NA	NA
**surgery**	only surgical margins negative	730	0.62	**5.3e-05**	0.54	**1.1e-06**	0.68	**0.012**
**radiotherapy**	no	276	0.81	0.28	0.72	0.11	0.64	0.059
	yes	73	1.72	0.1	1.64	0.13	0.64	0.18
**chemotherapy**	no	317	0.65	**0.015**	1.35	0.15	1.49	0.14
	yes	178	0.46	**0.0018**	1.45	0.13	0.58	**0.046**

HR, hazard ratio; AJCC. American Joint Committee on Cancer; OS, overall survival; FP, first progression; PPS, post progression survival; NA, not available data; *P* value less than 0.05 is shown in bold.

**Table 4:** Subgroup analysis on the correlation of *SMARCA1* expression and prognosis of gastric cancer cases.

Factor	Subgroup	Sample size	OS		FP		PPS	
			HR	*P*	HR	*P*	HR	*P*
**Gender**	Female	244	1.4	0.086	1.73	**0.031**	1.42	0.098
	Male	566	1.59	**8.2e-04**	1.6	**0.0024**	1.16	0.31
**Stage**	Stage 1	69	3.1	**0.039**	2.6	0.1	4.48	**0.0498**
	Stage 2	145	2.28	**0.0057**	2.52	**0.0023**	2.01	**0.036**
	Stage 3	319	1.66	**7.7e-04**	1.75	**0.013**	1.44	0.088
	Stage 4	152	1.26	0.25	1.37	0.14	0.76	0.23
**Stage t**	t1	14	NA	NA	NA	NA	NA	NA
	t2	253	1.99	**0.0013**	2.28	**6.5e-05**	1.7	**0.019**
	t3	208	1.3	0.16	1.4	0.099	1.42	0.099
	t4	39	1.94	0.14	1.62	0.26	2.33	0.067
**Stage n**	n0	76	3.22	**0.0051**	3.4	**0.0043**	6.13	**8.7e-04**
	n1	232	1.79	**0.0053**	1.88	**0.0013**	1.54	0.095
	n2	129	0.82	0.42	0.8	0.31	1.46	0.11
	n3	76	1.77	**0.048**	2.17	**0.019**	1.39	0.29
**Stage m**	m0	459	1.67	**0.001**	1.69	**4.6e-04**	1.63	**0.0076**
	m1	58	1.67	0.096	1.75	0.063	0.5	0.091
**HER2**	negative	641	1.51	**9.6e-04**	1.73	**7.9e-05**	1.52	**0.011**
	positive	424	1.66	**0.0017**	1.45	0.068	0.66	**0.025**
**Lauren classification**	Instestinal	336	1.68	**0.0011**	1.76	**0.0014**	1.56	0.096
	Diffuse	248	1.5	**0.049**	1.83	**0.0085**	1.58	0.053
	Mixed	33	4.88	0.09	4.68	0.1	NA	NA
**Differentiation**	Poorly	166	1.41	0.14	0.69	0.18	0.45	**0.02**
	Moderately	67	1.68	0.11	1.61	0.13	1.82	0.21
	Well	32	2.38	**0.045**	NA	NA	NA	NA
**Treatment**	Surgery alone	393	1.67	**0.0014**	1.67	**3.3e-04**	1.6	**0.0078**
	5-Fu based adjuvant	157	0.62	**0.0082**	0.7	0.054	0.61	**0.0069**
	Other adjuvent	80	0.63	0.34	2.16	0.12	0.66	0.38
**Perforation**	No	169	1.48	0.086	1.33	0.18	1.48	0.19
	yes	4	NA	NA	NA	NA	NA	NA

HR, hazard ratio; OS, overall survival; FP, first progression; PPS, post progression survival; HER2, human epidermal growth factor receptor-2; NA, not available data; *P* value less than 0.05 is shown in bold.

**Table 5:** Subgroup analysis on the correlation of *SMARCA1* expression and prognosis of liver cancer cases.

Factor	Subgroup	Sample size	OS		PFS		RFS		DSS	
			HR	*P*	HR	*P*	HR	*P*	HR	*P*
**Stage**	Stage 1	171	1.55	0.24	1.84	0.062	0.59	0.072	2.66	0.11
	Stage 2	86	1.9	0.11	1.49	0.21	1.98	**0.048**	3.53	**0.016**
	Stage 3	85	0.63	0.14	2.31	**0.025**	1.45	0.23	1.55	0.22
	Stage 4	5	NA	NA	NA	NA	NA	NA	NA	NA
**Grade**	Grade 1	55	0.21	**3.9e-04**	0.44	0.069	0.38	0.082	0.35	0.087
	Grade 2	177	1.6	0.074	0.91	0.67	0.78	0.38	1.9	0.054
	Grade 3	122	1.4	0.33	1.98	**0.022**	2.07	**0.024**	1.79	0.14
	Grade 4	12	NA	NA	NA	NA	NA	NA	NA	NA
**AJCC_T**	T1	181	1.66	0.17	1.89	0.051	0.64	0.11	3.07	0.057
	T2	94	1.89	0.092	1.48	0.19	1.79	0.079	2.94	**0.021**
	T3	80	0.63	0.16	2.04	0.06	1.43	0.36	2.14	0.21
	T4	13	NA	NA	NA	NA	NA	NA	NA	NA
**Gender**	Female	121	2.24	**0.0045**	2.37	**0.0015**	2.5	**0.0044**	3.46	**9.3e-04**
	Male	250	0.58	**0.023**	0.66	**0.025**	0.61	**0.014**	0.65	0.14
**Vascular invasion**	None	205	1.44	0.18	1.36	0.23	0.75	0.27	1.92	0.095
	micro	93	1.72	0.17	1.3	0.41	0.8	0.48	3.83	0.16
	macro	16	NA	NA	NA	NA	NA	NA	NA	NA
**Race**	White	184	1.37	0.19	1.36	0.16	1.21	0.42	1.89	**0.024**
	Asian	158	0.58	0.08	1.5	0.15	1.41	0.25	0.59	0.2
	Black or African american	17	NA	NA	NA	NA	NA	NA	NA	NA
**Sorafenib treatment**	treated	30	5.06	**0.0035**	1.93	0.099	1.74	0.24	5.06	**0.0035**
**Alcohol consumption**	Yes	117	0.56	0.075	0.67	0.14	0.56	0.055	0.77	0.47
	none	205	1.24	0.38	1.65	**0.037**	1.73	**0.043**	2.07	**0.023**
**Hepatitis virus**	Yes	153	0.64	0.2	0.64	0.074	0.68	0.14	1.5	0.35
	none	169	1.57	0.073	1.81	**0.012**	1.51	0.11	2.09	**0.015**

HR, hazard ratio; AJCC. American Joint Committee on Cancer; OS, overall survival; PFS, progress free survival; RFS, relapse free survival; DSS, disease specific survival; NA, not available data; *P* value less than 0.05 is shown in bold.

### 3.3 Genetic alteration analysis

**Fig 8:**
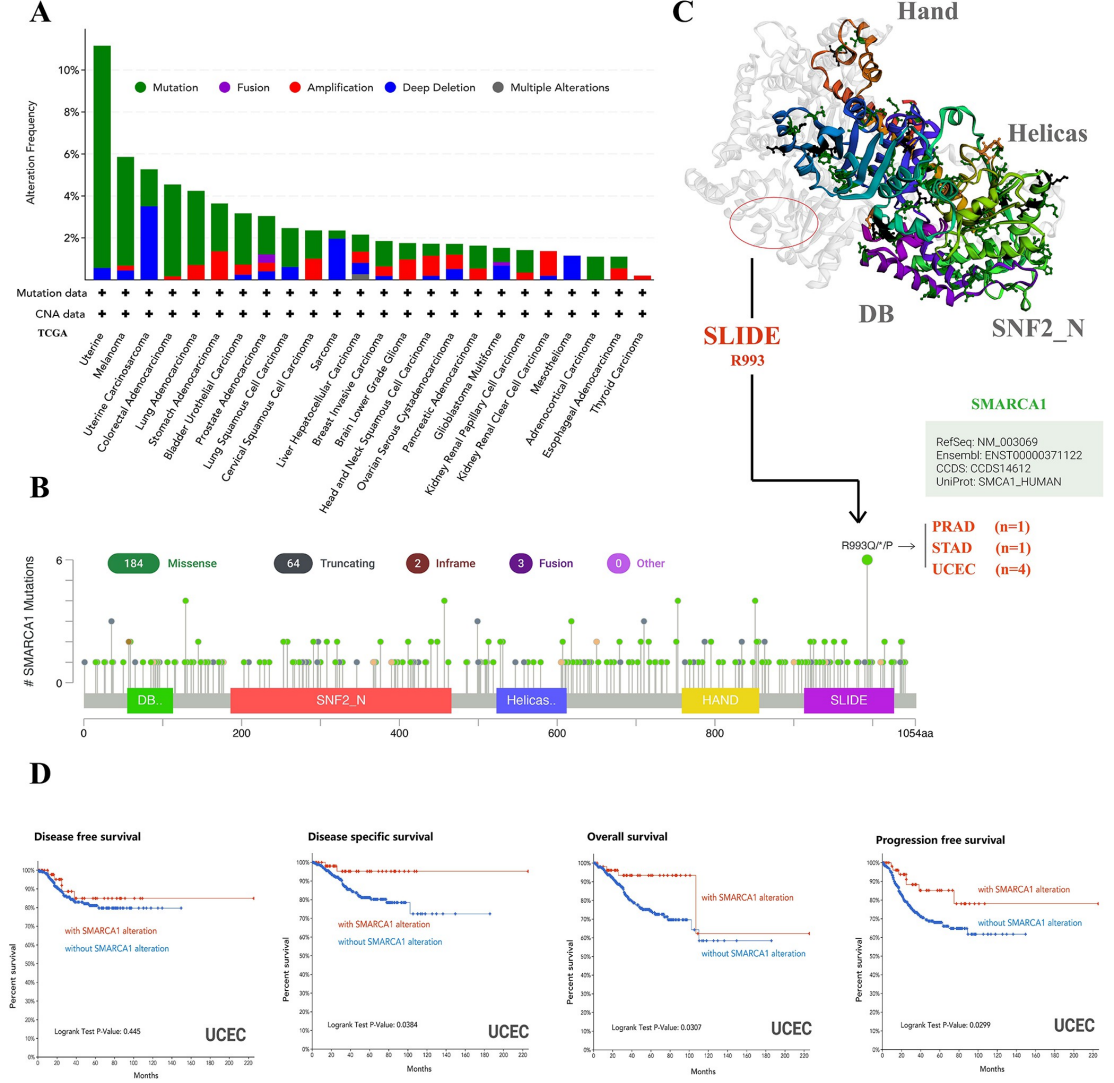
Genetic variation features of SMARCA1 in different cancers based on TCGA via cBioPortal. (A) The alteration frequency and types among diverse cancers; (B) Potential sites of mutation; (C) mutation site with highest frequency (R993Q/*/P) in the 3D version of SMARCA1 and related carcinomas; (D) Prognosis analysis between SMARCA1 alteration and OS, DSS, DFS, and PFS of uterine corpus endometrial carcinoma (UCEC) by the cBioPortal. *P < 0.05.

Using the cBioPortal tool, we obtained the genetic variation status of SMARCA1 across different cancers in the TCGA database. The highest alteration frequency of SMARCA1 (> 10%) appeared in uterine cancers with “mutation” as the main component. “Deep deletion” showed an alteration frequency of ~4% in the uterine carcinosarcoma as the primary type (Fig 8A). Although “amplification” was predominant in renal clear cell carcinoma and “deep deletion” in mesothelioma, it is inconsequential due to having an alteration frequency < 2% (Fig 8A). Detailed information of mutation types, sites and frequencies of SMARCA1 variation is shown in Fig 8B. Missense alteration of SMARCA1 was the most common form and R993Q/*/P mutation in the SLIDE domain was detected in 4 cases of UCEC, 1 case of PRAD and 1 case of STAD (Fig 8B), which may result in frame-shift mutation of the SMARCA1 gene, translation from R (arginine) to Q (glutamine)/P (proline) at the 993 site of SMARCA1 protein and proceeding protein truncation. Although the complete structure of the SMARCA1 protein is unclear, the potential position of the R993 site can still be speculated in the 3D diagram (Fig 8C).

We assessed the link between SMARCA1 gene variation and clinical prognosis of different cancers. As presented in Fig 8D, UCEC patients with SMARCA1 mutation had better OS, DSS and PFS (all *P* < 0.05) but worse DFS (*P* = 0.445), compared to the control.

### 3.4 DNA methylation analysis

**Fig 9:**
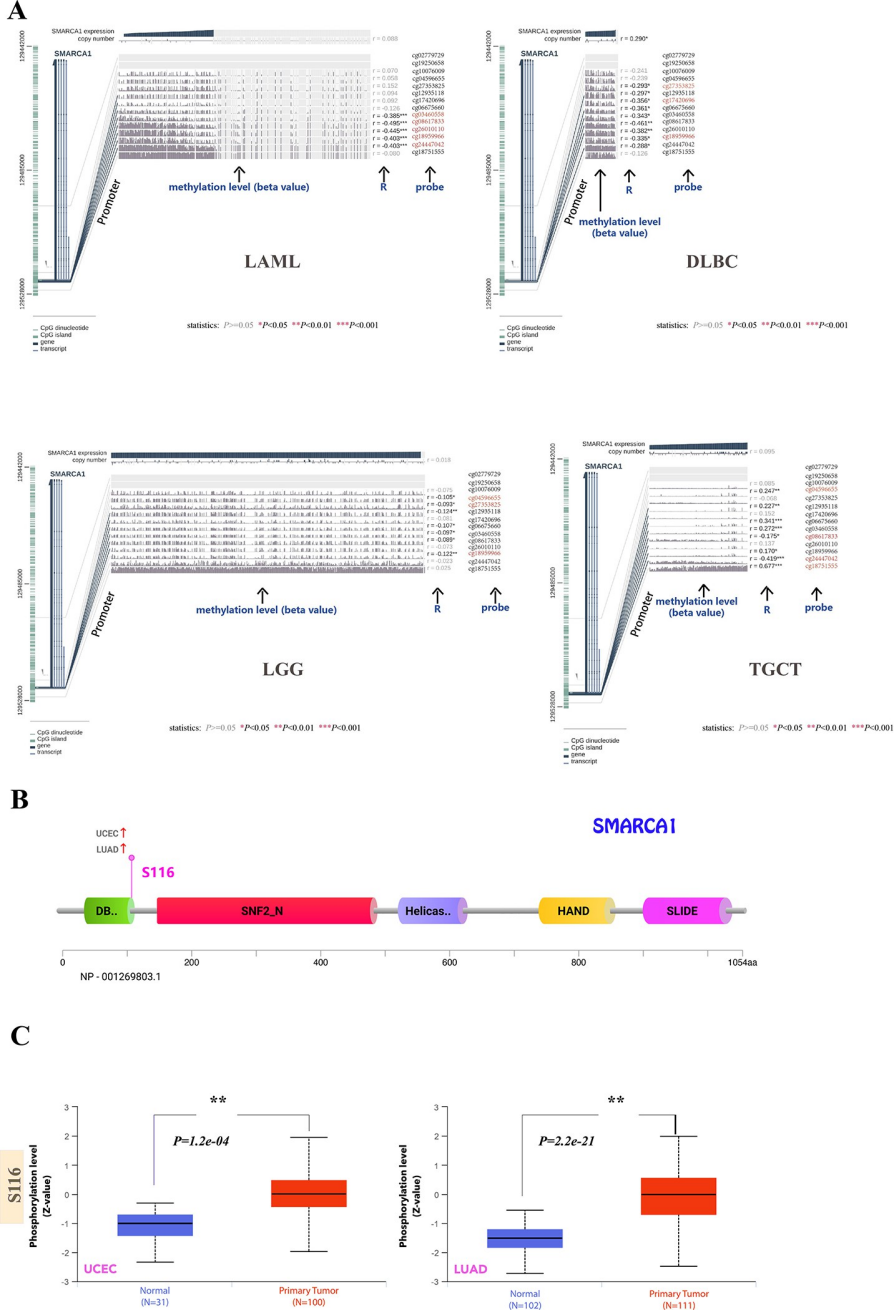
DNA methylation and phosphorylation analysis of SMARCA1 expression in different cancers of TCGA. (A) The correlation analysis between SMARCA1 DNA methylation and multiple cancers (LAML, LGG, DLBC, TGCT) of diverse probes was displayed with beta value, Pearson correlation coefficients (R) and Benjamini-Hochberg-adjusted *P*-value. (B) The structural diagram of SMARCA1 protein with positive phosphoprotein sites and cancers related. (C) comparison of phosphoprotein level between tumor and normal tissues on UCEC and LUAD.

Using MEXPRESS, we looked into the potential relationship between SMARCA1 DNA methylation and oncogenesis in different neoplasms. Despite the insufficient methylation data, we could still observe some significant differences in several probes. With respect to LAML, we found a significant negative correlation at multiple probes (e.g., cg03460558, cg08617833, etc.) of the promoter region (all *P* < 0.001) (Fig 9A). Similarly, negative correlation was observed at probes (cg27353825, cg17420696, cg18959966) of non-promoter region in DLBC (*P* < 0.05) and in probes (cg04596655, cg27353825, cg18959966) of non-promoter region in LGG (*P* < 0.05) (Fig 9A). Interestingly, we not only found negative correlation in probes (cg08617833, cg24447042) of promoter region, but also positive correlation in probes (cg04596655, cg18751555) of non-promoter region appearing in TGCT (*P* < 0.05) (Fig 9A). Due to limited research on the field, we were unable to further verify the relationship between expression and DNA methylation of SMARCA1.

### 3.5 Protein phosphorylation analysis

Based on the CPTAC database, we investigated the differences in SMARCA1 protein phosphorylation between primary tumors and normal tissues. Owing to limited data, only UCEC and LUAD associated with S116 site were analyzed. Findings of correlation between SMARCA1 phosphorylation and tumor are summarized in Fig 9B. S116, located in the DB domain of SMARCA1 displays higher phosphorylation levels in primary tumor tissues compared to normal tissues for UCEC and LUAD (all *P* < 0.001) (Fig 9C). Reference parameters for the CPTAC-identified phosphorylation site of SMARCA1 using PhosphoNet database are listed in Table 6, however, these are not backed with experimental data. These results indicate that further investigation of SMARCA1 phosphorylation is warranted.

**Table 6:** Analysis of CPTAC-identified phosphorylation sites of *SMARCA1* via the PhosphoNET database.

site	sequence	experimentally confirmed^#^	hydrophobicity	p-site similarity score	maximum kinase specificity	sum kinase specificity score	conservation score
S116	SAQKSPTSP	NA	-0.847	-56.9	676	23,731	23.0

^*#*^The PMID (PubMed Unique Identifier) information of the publication was provided; NA, not available.

### 3.6 Tumor-related immune infiltration analysis

**Fig 10:**
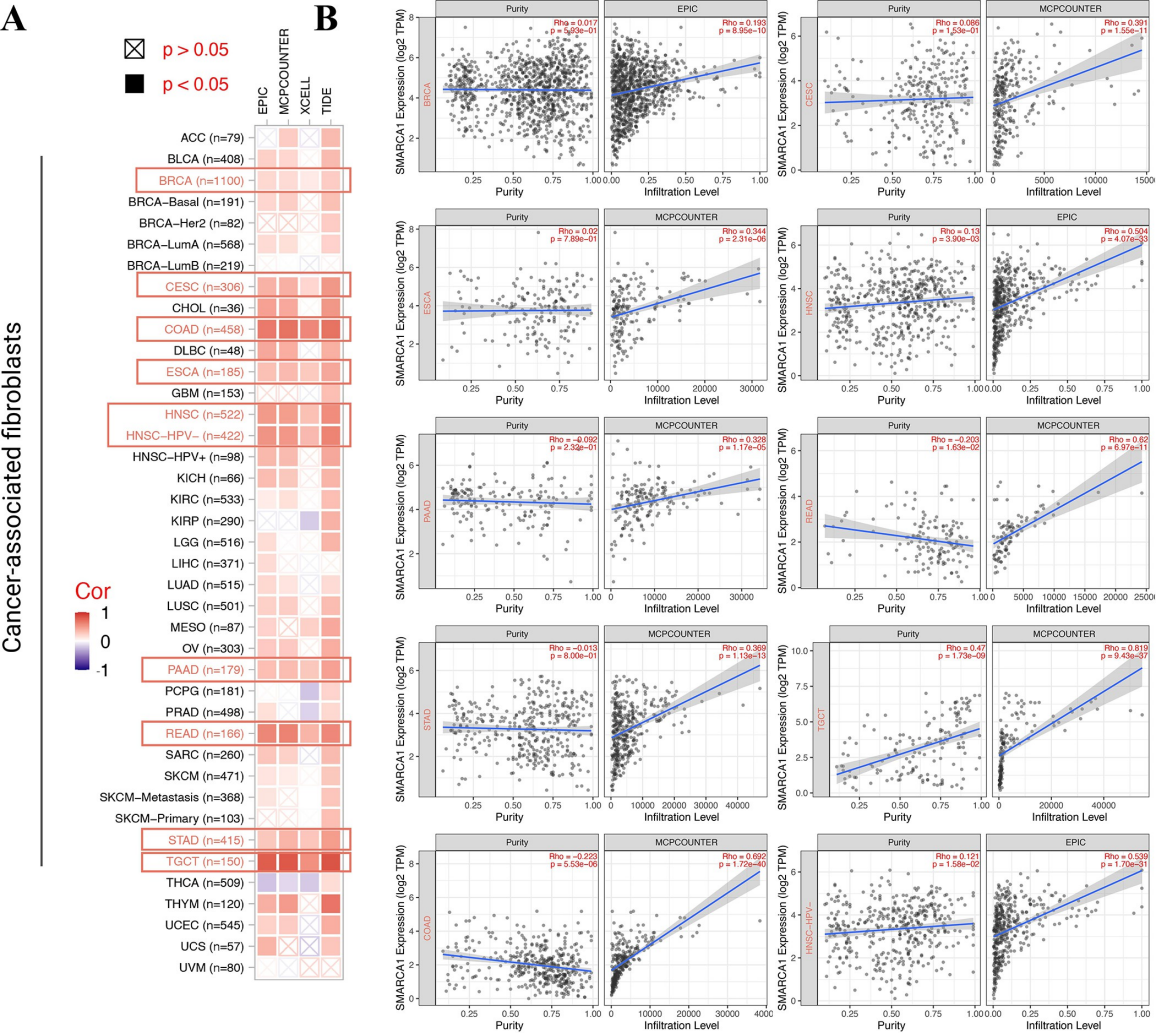
Correlation analysis between SMARCA1 expression and cancer-associated fibroblasts. (A) Heatmap of correlation between SMARCA1 and the tumor-related immune infiltration level of cancer-associated fibroblasts among all cancers in TCGA with diverse of algorithms; (B) correlation analysis of BRCA, CESC, COAD, ESCA, HNSC, HNSC-HPV, PAAD, READ, STAD and TGCT.

**Fig 11:**
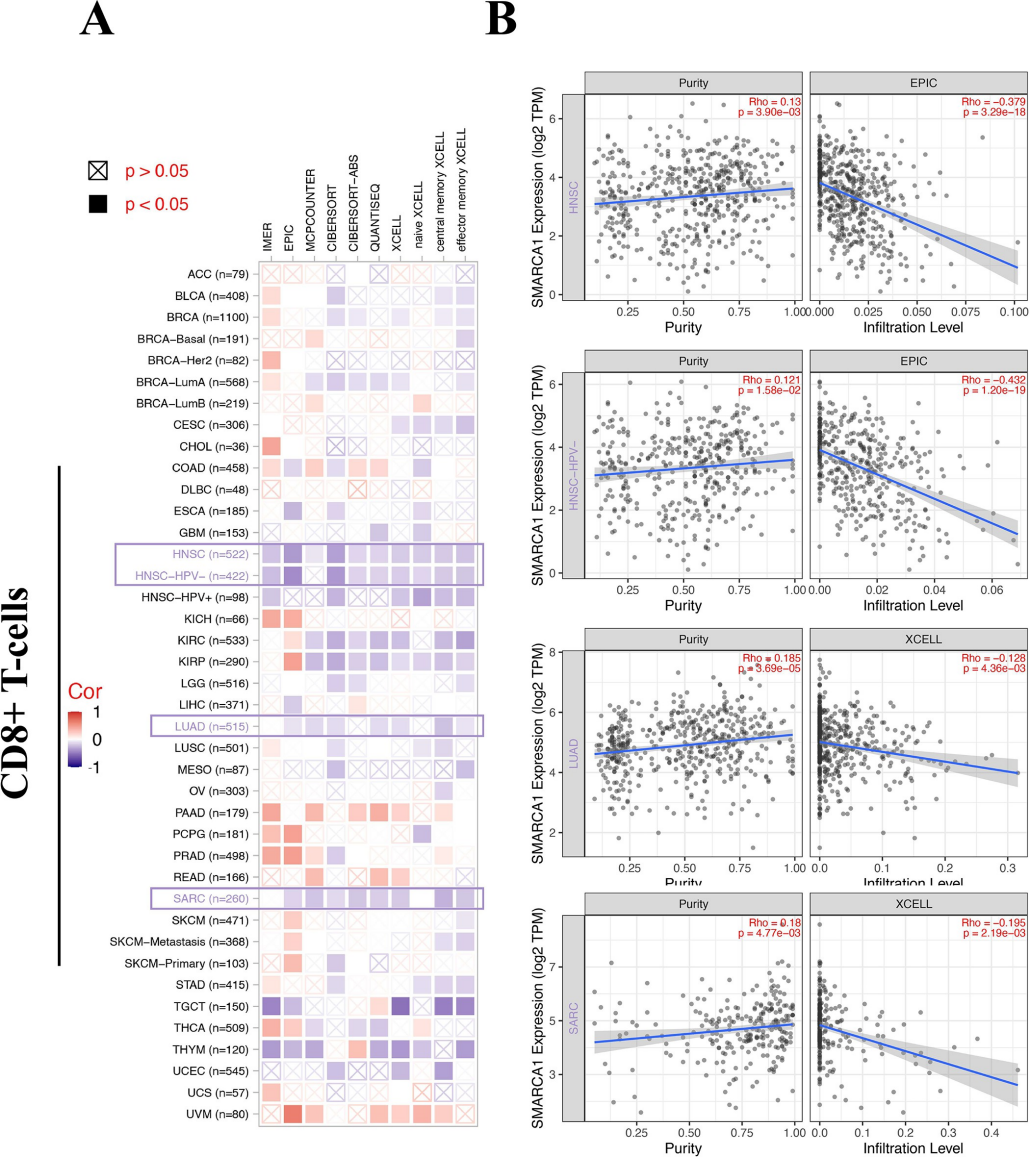
Correlation analysis between SMARCA1 expression and CD8^+^ T-cells infiltration. (A) Heatmap of correlation between SMARCA1 and the tumor-related immune infiltration level of CD8^+^ T-cells among all cancers in TCGA with diverse of algorithms; (B) correlation analysis of HNSC, HNSC-HPV, LUAD and SARC.

Tumor-related immune infiltration and its association with cancer pathogenesis, progression and metastasis gained increasing interest and focus [43, 44]. Cancer-associated fibroblasts were considered a significant component of the tumor-immunity microenvironment, exhibiting functions such as matrix remodeling, signal interaction and immune infiltration interfering [45–48]. Therefore, we used QUANTISEQ, TIMER, MCPCOUNTER, XCELL, CIBERSORT, CIBERSORT-ABS, TIDE and EPIC algorithms to assess the correlation between SMARCA1 expression and the immune infiltration level of different cell types in various of TCGA cancers. We found a statistically significant positive relationship between SMARCA1 and the estimated value of cancer-associated fibroblasts infiltration for BRCA, CESC, COAD, ESCA, HNSC, HNSC-HPV^-^, PAAD, READ, STAD and TGCT (Fig 10A, *P* < 0.05) based on all the algorithms. In contrast, a negative relationship was observed between CD8+ T-cells infiltration and SMARCA1 expression for HNSC, HNSC-HPV^-^, LUAD and SARC (Fig 11A, *P* < 0.05) with most algorithms [24, 49, 50]. We also produced scatterplots of the above cancers with the most effective algorithm (Figs 10B, 11B).

**Fig 12:**
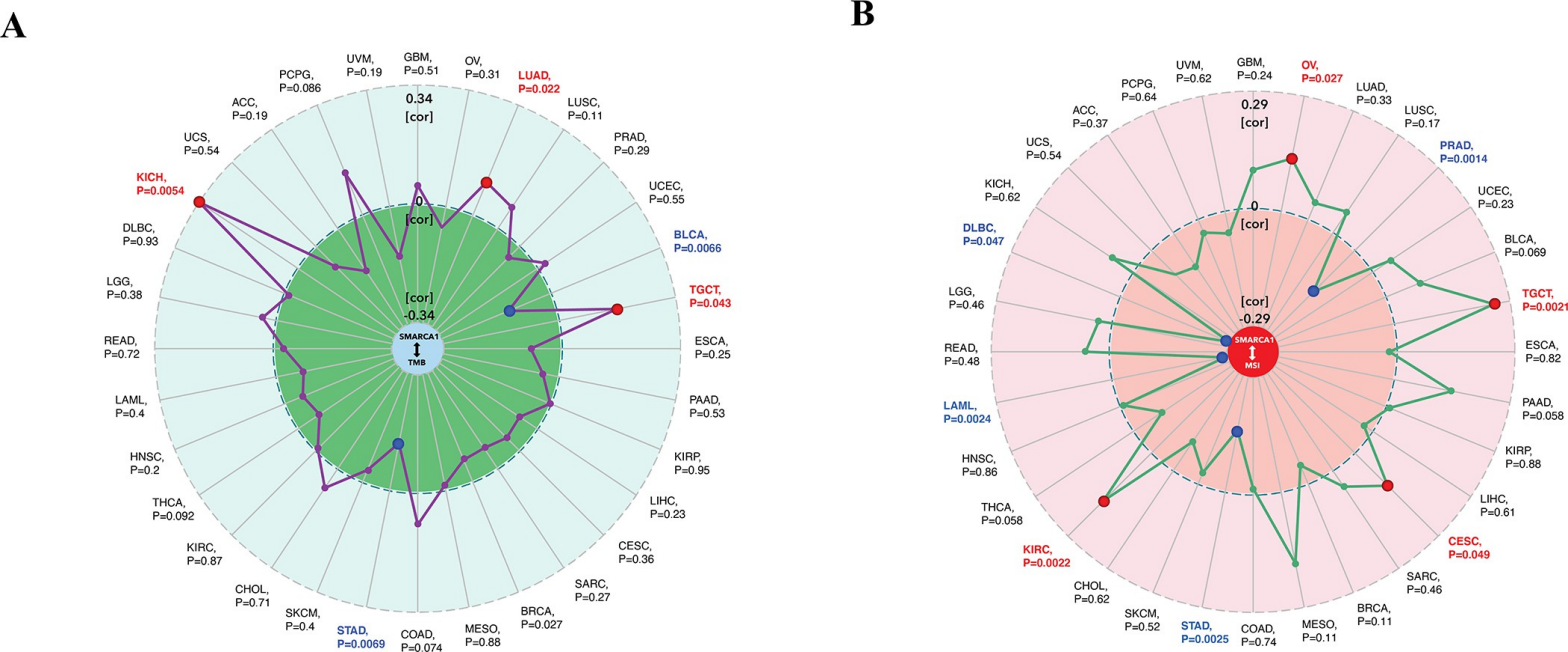
Correlation analysis between SMARCA1 and TMB/MSI. (A) Gene-immune analysis of the potential correlation between SMARCA1 expression and TMB across all cancers in TCGA. The partial correlation values (cor) of +0.34 and -0.34 are marked. (B) Gene-immune analysis of the potential correlation between SMARCA1 expression and MSI across all cancers in TCGA. The partial correlation values (cor) of +0.29 and -0.29 are marked. *P-value < 0.05. TMB: tumor mutational burden; MSI: microsatellite instability.

Furthermore, we explored the relationship between SMARCA1 and tumor mutational burden (TMB) / microsatellite instability (MSI), which were used for the prediction of immunotherapy efficacy [51, 52]. All the cancers in TCGA were analyzed and results presented in Fig 12. SMARCA1 expression was positively correlated with TMB for KICH (*P* = 0.0054), LUAD (*P* = 0.022) and TCGT (*P* = 0.043) but negatively for BLCA (*P* = 0.0066) and STAD (*P* = 0.0069) (Fig 12A). We also observed a positive relationship between SMARCA1 and MSI for OV (*P* = 0.027), TGCT (*P* = 0.0021), CESC (*P* = 0.049) and KIRC (*P* = 0.0022), but negative for PRAD (*P* = 0.0014), STAD (*P* = 0.0025), LAML (*P* = 0.0024) and DLBC (*P* = 0,047) (Fig 12B). This aspect needs further research.

### 3.7 Enrichment analysis of SMARCA1-related genes

**Fig 13:**
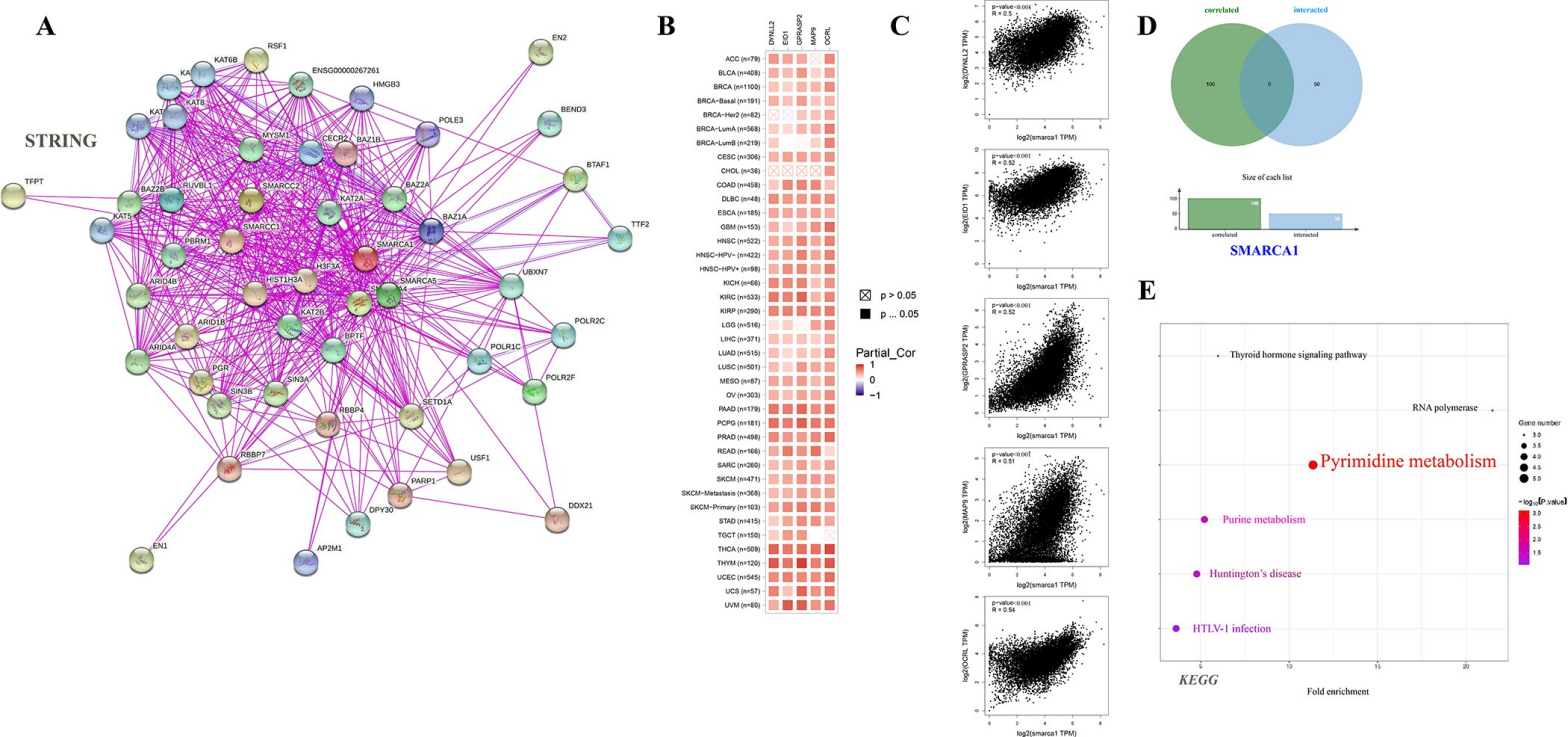
SMARCA1-related gene enrichment analysis. (A) SMARCA1-related functional proteins association networks with experimental determination via STRING database; (B) The heatmap of correlation analysis between SMARCA1 and the selected targeting genes of top 100 SMARCA1-related genes in all TCGA cancers; (C) The detailed correlation analysis between SMARCA1 and corresponding genes, including DYNLL2, EID1, GPRASP2, MAP9, and OCRL; (D) cross-analysis of the SMARCA1 correlated and interacted genes; (E) KEGG pathway analysis of SMARCA1-related genes.

For an in-depth exploration of the SMARCA1 molecular mechanism in oncogenicity, we tried to sift out the targeted SMARCA1-interacted genes and SMARCA1-correlated genes for a set of signal pathway enrichment analyses. We acquired 50 SMARCA1-binding proteins certificated by experimental evidence via the STRING instrument and constructed an interaction network diagram of them (Fig 13A) [38]. We then used the “similar genes detection” module of GEPIA2 to obtain the top 100 SMARCA1 expression-correlated genes based on tumor types in TCGA. Generally, the top 5 selected genes exhibited a substantial positive reverence to SMARCA1 in most cancer types in a heatmap (Fig 13B) [24, 49, 50]. Scatterplots showed a strong positive correlation between SMARCA1 expression and dynein light chain 2 (DYNLL2) (R = 0.50), empfindlicher im dunkelroten licht1 (EID1) (R = 0.52), G protein-coupled receptor associated sorting protein 2 (GPRASP2) (R = 0.52), microtubule-associated protein 9 (MAP9) (R = 0.51), oculocerebrorenal syndrome of Lowe (OCRL) (R = 0.54) genes (all P < 0.001) (Fig 13C).

**Fig 14:**
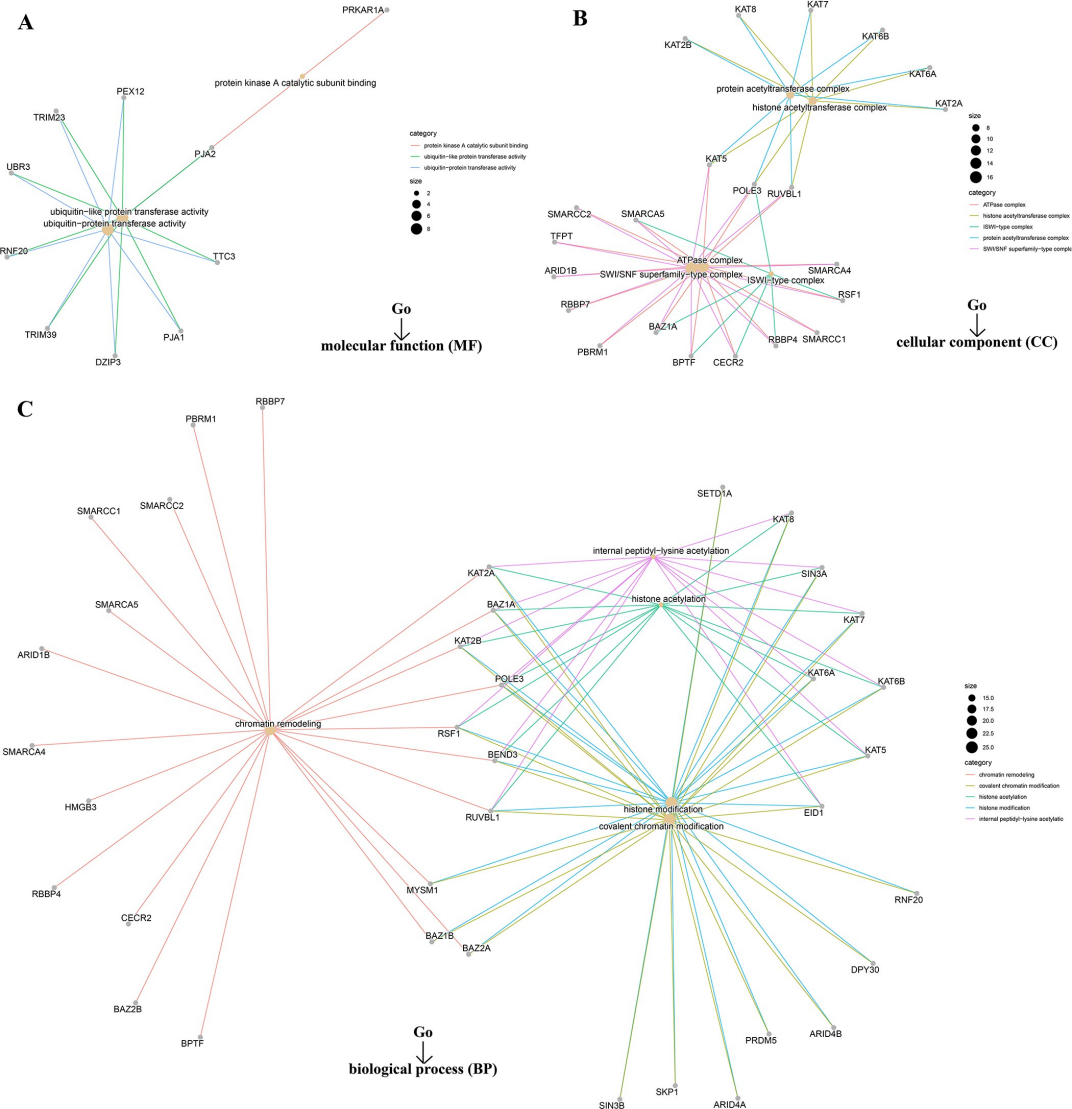
Go analyses of SMARCA1-related genes in different cancers. (A) The cnetplots for the molecular function (MF) data; (B) The cnetplots for the cellular component (CC) data; (C) The cnetplots for the biological process (BP) data.

However, we failed to screen out the common member genes from an intersection analysis of the two groups above (Fig 13D). We conducted KEGG and Go enrichment analyses by combining the two datasets. The KEGG plot suggested that “pyrimidine metabolism” and “purine metabolism” might be the main pathways involved in SMARCA1 oncogenicity (Fig 13E) [40, 41, 53–55]. The Go enrichment analyses further predicted the intimate connection between most of these genes and the pathway or biological progress of chromatin remodeling, including ubiquitin-protein transferase activity, SWI/SNF superfamily-type complex, histone acetyltransferase complex, covalent chromatin modification and others (Fig 14).

### 3.8 Diagnostic capability analysis

Through the comprehensive analysis, we discovered that SMARCA1 had good diagnostic performance on 29 cancers, especially on OV (AUC = 0.981), LAML (AUC = 0.935), DLBC (AUC = 0.970), CESC (AUC = 0.846), READ (AUC = 0.802), COAD (AUC = 0.807), LGG (AUC = 0.938), THYM (AUC = 0.992), PAAD (AUC = 0.898), GBM (AUC = 0.838) and TGCT (AUC = 0.910) (S5 Fig).

## 4. Discussion

As reported in prior studies, the main functions of SMARCA1 protein are focused on chromatin structure alteration and transcriptional activation across a wide range of species [6–11, 56, 57]. Our study demonstrated structure conservation of SMARCA1 translation products across several species through the “HomoloGene” database and phylogenetic tree analysis, indicating that similar biological mechanisms of SMARCA1 might operate in the regular physiological activity across each species. Apart from the potential correlation between SMARCA1 and different diseases, especially cancer [15–19] were revealed by numerous studies. Whether SMARCA1 could play an essential role in the carcinogenesis of different cancers through established or undiscovered molecular mechanisms is yet to be determined. At the time of writing, to the best of our knowledge, no pan-cancer analysis of SMARCA1 has been reported in published literature. Hence, we thoroughly evaluated the SMARCA1 gene across all cancers based on several databases including TCGA, GEO, CPTAC and more. The molecular characteristic, survival prognosis, genetic mutation, DNA methylation, protein phosphorylation, immune infiltration, signal pathway and diagnostic value were analyzed.

The expression of SMARCA1 varied with each tumor, and so did diagnosis and prognosis, which probably indicates different tumorigenic mechanisms existed in different cancers. SMARCA1 was previously reported to play a vital role in the maturation of midbrain dopaminergic (mDA) neurons [58] and is related with multiple mechanisms in neurogenesis or schizophrenia [15]. Although it was considered to be correlated with neurodevelopmental disorders, t a lack of studies specially focused on the brain and central neural system (CNS) cancers. This study attempted to use several tools to assess the correlation between SMARCA1 expression and CNS malignant tumors. Despite our failure to find significant differences based on data in the TIMER2 database, we found that SMARCA1 expression was much higher in tumor tissues than in normal tissues in GBM and LGG based on the GEPIA2 data (combined TCGA and GTEx database) (*P* < 0.001). This inconsistency might be due to the insufficient normal tissues for control in TIMER2. Up on further analysis of prognosis, we found that higher expression of SMARCA1 was correlated with poor OS (*P* = 0.036) and DFS (*P* = 0.035) in LGG, but was not statistically significant in GBM (Fig 5). Similar results were found by OncoLnc (http://www.oncolnc.org), with Cox regression survival analysis on LGG (Cox coefficient = 0.373, *P* = 1.10e-04) and GBM (Cox coefficient = -0.075, *P* = 4.20e-01). Additionally, the correlation between SMARCA1 and brain and CNS cancer was verified again via the Oncomine database (P = 7.64e-06) (Fig 3B). Great diagnostic value of SMARCA1 was observed in both cancers (S5 Fig). We observed an unexpected association between reduced DNA methylation levels at three sites in the non-promoter region and high expression of SMARCA1 in LGG (Fig 9A). Consequently, the potential mechanisms of DNA methylation and related upstream or downstream pathways remain to be explored and other CNS tumors deserve to be included in any future investigations.

For lung carcinoma, we used TIMER2 to analyze the datasets of TCGA-LUAD (n = 515) and TCGA-LUSC (n = 501) projects and obtained completely opposite results. High SMARCA1 expression in tumor tissues of LUAD was related to poor OS (*P* = 0.009) and DFS (*P* = 0.037) (Fig 5). Nevertheless, low SMARCA1 expression in tumor tissues of LUSC conversely correlated with a better DFS (*P* = 0.013). Furthermore, we analyzed lung adenocarcinoma (n = 865) and squamous cell carcinoma (n = 675) cases from all cohorts, revealing that low SMARCA1 expression was correlated with poor OS, FP and PPS in LUAD, however high expression was associated with poor FP in LUSC (Table 3). The inconsistent in LUAD might due to the sample size and as a result, the opposite role of SMARCA1 in patient prognosis with different lung cancer histopathology needs further investigation with more extensive sample size studies.

SMARCA1 expression has been found to be correlated with liver cancer in a few studies. Therefore, we conducted a series of statistical analyses to examine this relationship. Based on TIMER2 analysis, SMARCA1 expression was high in tumor tissues of LIHC but low in cholangiocarcinoma (CHOL). However, there were no significant differences between SMARCA1 expression and prognosis in liver cancer based on GEPIA2, OncoLnc, and Kaplan-Meier plotter approaches. Although it could be affected by heterogeneity, model, effect size, sample size, bias, etc., the meta-analysis indicated a potentially high risk of poor prognosis in liver cancer with elevated SMARCA1 expression to (*P* = 0.021). After analyzing the subgroups of LIHC, we found that female patients with high SMARCA1 expression had poor OS, PFS, RFS and DSS (Table 5), indicating that gender might be a potential risk factor. Although big data cannot give sufficient evidence to support the tumor oncogenic role of SMARCA1 in the prognosis of LIHC as yet, further studies might influence the results.

Down-regulated SMARCA1 might contribute to DNA injury, growth restriction and tumor cell apoptosis in breast cancer [59] were previously reported, without providing prognosis analysis and other bioinformation. Our study discovered that a low expression of SMARCA1 was correlated with poor OS, RFS and DMFS in breast cancer based on large sample sizes. However, SMARCA1 protein expression was unexpectedly elevated in tumor tissues. The findings may have been affected by the small sample size of the control group. Extensive sample size studies are needed. Many potential mechanisms intervening the process of RNA to protein should also be considered. Although no significant independent risk factors in subgroups were identified (Table 1), low SMARCA1 expression was generally considered a potential risk indicator related to the poor prognosis of BRCA.

For gastrointestinal tumors, up-regulated SMARCA1 expression was considered to be correlated with colorectal and gastric cancer through different pathways [17, 18]. Interestingly, higher SMARCA1 expression was associated with better DFS based on the GEPIA2 database regardless of the low expression of SMARCA1 in tumor tissues of COAD verified by TIMER2 and Oncomine. Since there were more normal tissues in the GEPIA2 control sample, this may have introduced inconsistencies in the results. Unlike COAD, we observed a consistent relationship between elevated SMARCA1 expression and poor prognosis of STAD across the different databases which indicates that SMARCA1 might be an independent risk factor for STAD. Consequently, the cause of this inconsistency in COAD remains ambiguous.

A similar phenomenon was observed in ovarian cancer: transcriptional SMARCA1 expression was low in tumor tissues accompanied by a high protein expression, with the increased expression correlating with poor OS, PFS, and PPS of OV. These inconsistent conclusions might also be due to the different sample size, data resources and underlying interference mechanisms at the translation level. Chemotherapy regimens might play a critical role in the SMARCA1-related prognosis (Table 2). Although our study discovered that high expression of SMARCA1 is related to poor prognosis of OV, factors contributing to a difference in transcriptional and translational expression need further investigation.

Our analysis of genitourinary cancer achieved comparatively consistent conclusions. The poorer prognosis of KIRC, KIRP, KICH and UCEC were correlated with lower expression of SMARCA1, which was also down-regulated in tumor tissues, indicating that low SMARCA1 expression might be an independent risk factor for genitourinary. Due to insufficient TGCT data, we only evaluated SMARCA1 expression and DNA methylation. SMARCA1 expression levels were low in tumor tissues whereas results from the DNA methylation level analysis showed that two sites (cg08617833and cg24447042) in the promoter region were negatively correlated with SMARCA1 expression. However, another two sites (cg04596655 and cg18751555) in the non-promoter region were positively associated with SMARCA1 expression. This suggests that, some molecular biomarkers or pathways are involved in the progression of SMARCA1 expression and need further evaluation.

In our study, we first explored the potential association between SMARCA1 expression and TMB/MSI across all tumors in TCGA, which needs clinical evidence to verify and validate. We then combined the bioinformation on SMARCA1-associated components and correlated genes covering all cancers for a chain of enrichment analyses to identify the critical role of “pyrimidine/purine metabolism pathways”, “chromatin remodeling and modification” and “ubiquitin-protein transferase activity” in oncogenicity of carcinomas. Furthermore, applying multiple deconvolution algorithms helped us obtain a statistically significant positive correlation between SMARCA1 and cancer-associated fibroblasts in BRCA, CESC, COAD, ESCA, head and neck squamous cell carcinoma (HNSC), HNSC-HPV^-^, PAAD, READ, STAD and TGCT. Using the same methods, we obtained a statistically significant negative correlation between SMARCA1 and CD8^+^ T-cells immune-infiltration in the cancers of HNSC, HNSC-HPV^-^, LUAD and SARC. This indicated that SMARCA1 expression might play a critical role in certain cancers through unknown mechanisms.

We also estimated the correlation between SMARCA1 DNA methylation and protein phosphorylation concerning different neoplasms. Due to insufficient relevant studies on the subject matter, we only observed a significant correlation between DNA methylation in LAML, DLBC, LGG and TGCT and phosphorylation at the S116 site in LUAD and UCEC without subsequent analyses of other biological information. Although the information we revealed was limited, the study still indicated that DNA methylation was related to potential sites and types of carcinomas, providing a direction for further research to some extent.

There are several limitations in our study. First, the SMARCA1 gene was not at the frontier of research, this restricted the relevant studies on it. Little attention has been paid to some types of cancers, this has resulted in limited sample sizes for those cancers which has a negative impact on the findings. Secondly, although we used plenty of databases and tools to elaborate on the role of SMARCA1 on tumorigenesis in many cancers, some of them were still unwell defined, primarily attributing to data updates being out of sync or to each database performing a singular function. We did not conduct the experiments or analyze the clinical data to provide a more concrete basis for the conclusions owing to the massiveness of the data required to complete the experimentation. Such experimentation is not feasible for a single center. Furthermore, as a bioinformatic analysis, although the batch effect of samples and cross-platforms had been corrected by ComBat, the potential interference was common and difficult to completely eliminate. In addition, we did not conduct an in-depth analysis of tumor-related immune infiltration and PPI due to space constrains. Although the analysis was insufficient, it is still meaningful in guiding future research direction to some extent.

SMARCA gene and its subsets play a dominant role in chromatin remodeling and restoration, whose deficiency might induce several malignancies [60]. Following this viewpoint, we integrated the big data of SMARCA1 gene expression, associated diagnosis, prognosis, genetic mutation, DNA methylation, protein phosphorylation, tumor-related immune infiltration, TMB and MSI across a spectrum of cancers to conduct a pan-cancer analysis and determine the potential correlations among them. Although there are some contradictory results, our study contributes to an initial understanding of the function and role of SMARCA1 in oncogenicity. It points out the direction of further prospective research that can provide much need insight and resolve outstanding inconsistencies.

## Supporting information

S1 Fig Correlation between SMARCA1 and different disorders and cancers. The reported pathogenic pathways mediated by SMARCA1 in different disorders and cancers are displayed in a graphic manner. The relevant references are included.
(TIF)

S2 Fig A flowchart for methods and materials. (PDF)

S3 Fig Structural characteristics of SMARCA1 in different species. (A) Genomic location of human SMARCA1; (B) Conserved domains of SMARCA1 protein among diverse species.
(TIF)

S4 Fig Phylogenetic tree of SMARCA1. We obtained the phylogenetic tree of SMARCA1 in different species via COBALT of NCBI. COBALT, constraint-based multiple alignment tool.
(TIF)

S5 Fig Diagnostic value of SMARCA1 among pan-cancer via TCGA + GTEx. SMARCA1 had different diagnostic abilities in different tumors. AUC, area under curve. * *P* < 0.05, ** *P* < 0.01, *** *P* < 0.001.
(PDF)

S1 File (ZIP)

## Abbreviations

ISWI: Imitation switch
TCGA: The cancer genome atlas
GTEx: Genotype-tissue expression
SMARCA1: SWI/SNF-related, matrix-associated, actin-dependent regulator of chromatin, subfamily A, member1
DBINO: DNA-binding domain
HELICc: Helicase superfamily c-terminal domain
SANT: ’SWI3, ADA2, N-CoR and TFIIIB’ DNA-binding domains
UCSC: University of California Santa Cruz
NCBI: National Center for Biotechnology Information
COBALT: Constraint-based multiple alignment tool
HPA: Human protein atlas
NX: Normalized expression
TIMER: The tumor immune estimation resource
GEPIA: Gene expression profiling interactive analysis
ACC: Adrenocortical Carcinoma
LGG: Lower Grade Glioma
FC: Fold change
TPM: Transcripts per million
CPTAC: Clinical proteomic tumor analysis consortium
RCC: Clear cell renal cell carcinoma
UCEC: Uterine corpus endometrial carcinoma
LUAD: Lung adenocarcinoma
OS: Overall survival
DFS: Disease-free survival
GEO: Gene expression omnibus
EGA: European genome-phenome archive
RFS: Relapse-free survival
DMFS: Distant metastasis-free survival
PPS: Post-progression survival
PFS: Progress-free survival
FP: First progression
DSS: Disease-specific survival
CI: Confidence interval
CNA: Copy number alteration
3D: Three-dimensional
MSI: Microsatellite instability
TMB: Tumor mutational burden
Cor: Correlation value
KEGG: Kyoto encyclopedia of genes and genomes
GO: Gene ontology
BP: Biological process
CC: Cellular component
MF: Molecular function
FANTOM5: Function annotation of the mammalian genome 5
BLCA: Bladder urothelial carcinoma
BRCA: Breast invasive carcinoma
COAD: Colon adenocarcinoma
KICH: Kidney chromophobe
KIRC: Kidney renal clear cell carcinoma
PRAD: Prostate adenocarcinoma
READ: Rectum adenocarcinoma
SKCM: Skin cutaneous carcinoma
THCA: Thyroid carcinoma
KIRP: Kidney renal papillary cell carcinoma
LUSC: Lung squamous cell carcinoma
CESC: Cervical squamous cell carcinoma and endocervical adenocarcinoma
CHOL: Cholangiocarcinoma
LIHC: Liver hepatocellular carcinoma
DLBC: Diffuse large B cell lymphoma
GBM: Glioblastoma multiforme
LAML: Acute myeloid leukemia
OV: Ovarian serous cystadenocarcinoma
PAAD: Pancreatic adenocarcinoma
TGCT: Testicular germ cell tumors
THYM: Thymoma
ESCA: Esophageal carcinoma
STAD: Stomach adenocarcinoma
UCS: Uterine carcinosarcoma
DYNLL2: Dynein light chain 2
EID1: Empfindlicher im dunkelroten licht1
GPRASP2: G protein-coupled receptor associated sorting protein 2
MAP9: Microtubule-associated protein 9
OCRL: Oculocerebrorenal syndrome of Lowe
mDA: Midbrain dopaminergic
HNSC: Head and neck squamous cell carcinoma
SARC: Sarcoma
ROC: Receiver operating characteristic
AUC: Area under curve
