# Supplementary figures and images for: A pan-cancer bioinformatic analysis of the carcinogenic role of SMARCA1 in human carcinomas
Source: PLoS One. 2022 Sep 20;17(9):e0274823. doi: 10.1371/journal.pone.0274823 (PMC9488775; doi:10.1371/journal.pone.0274823)

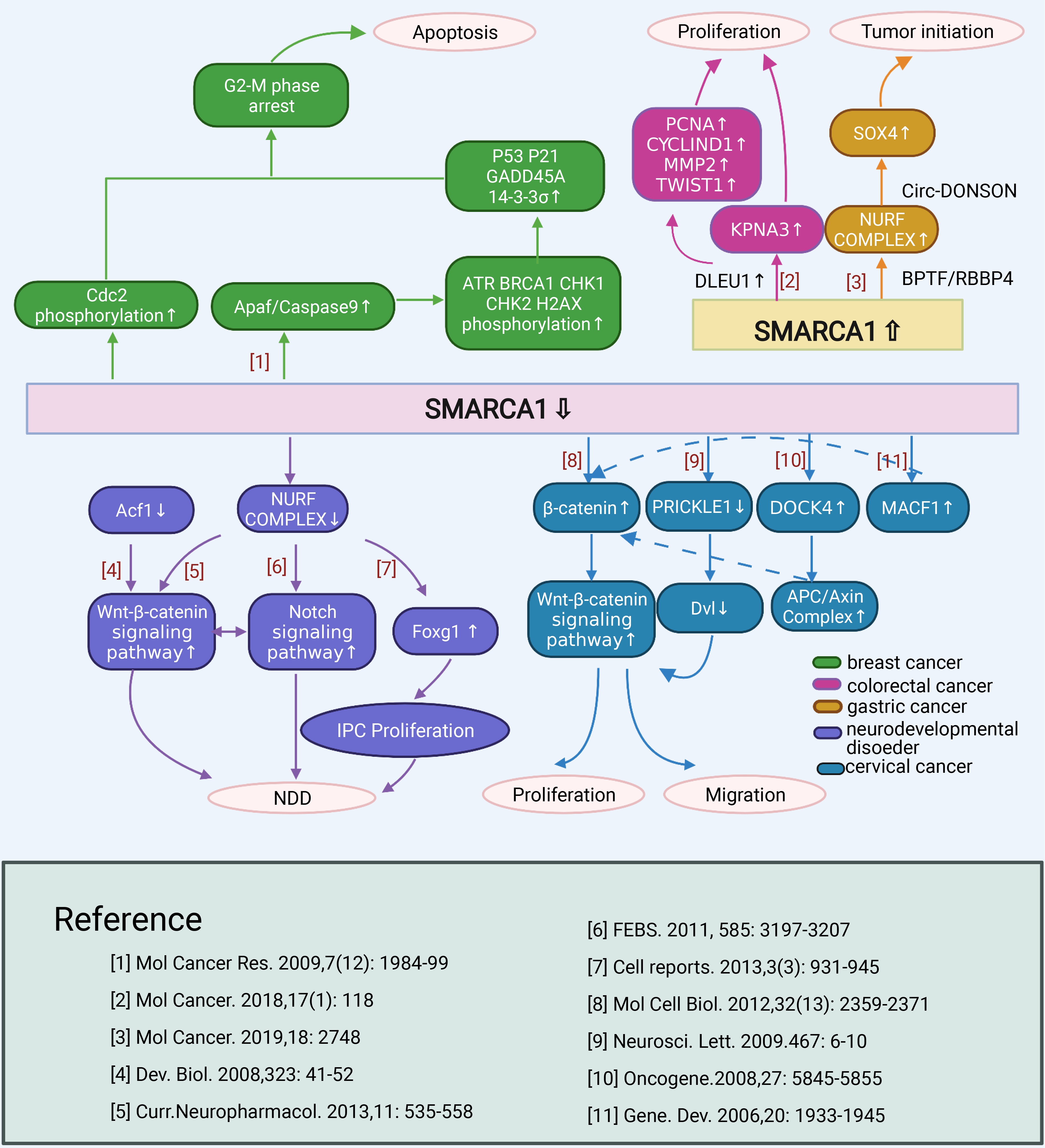

Supplement: S1 Fig — The reported pathogenic pathways mediated by SMARCA1 in different disorders and cancers are displayed in a graphic manner. The relevant references are included. (TIF) [file pone.0274823.s001.tif]

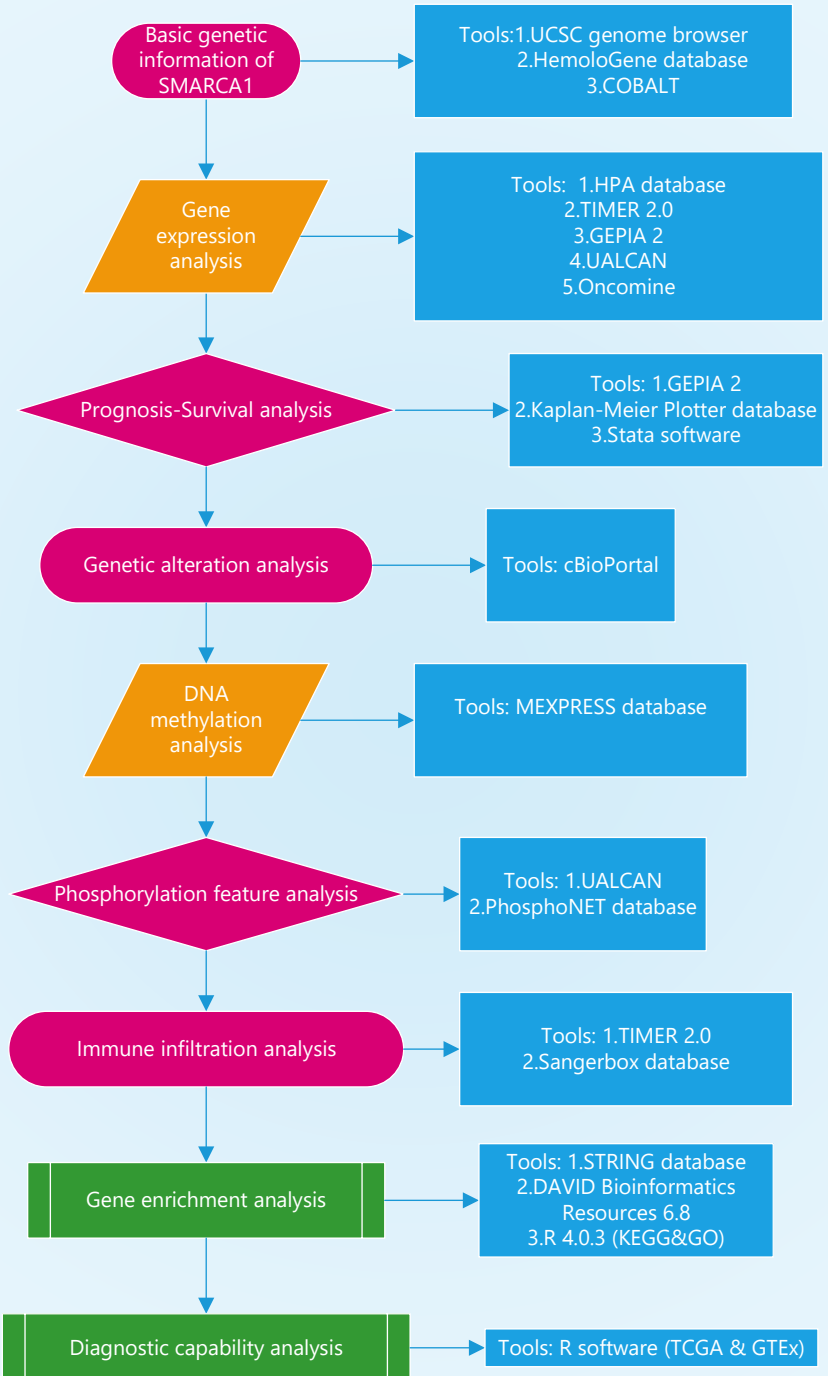

Supplement: S2 Fig — (PDF) [file pone.0274823.s002.pdf]

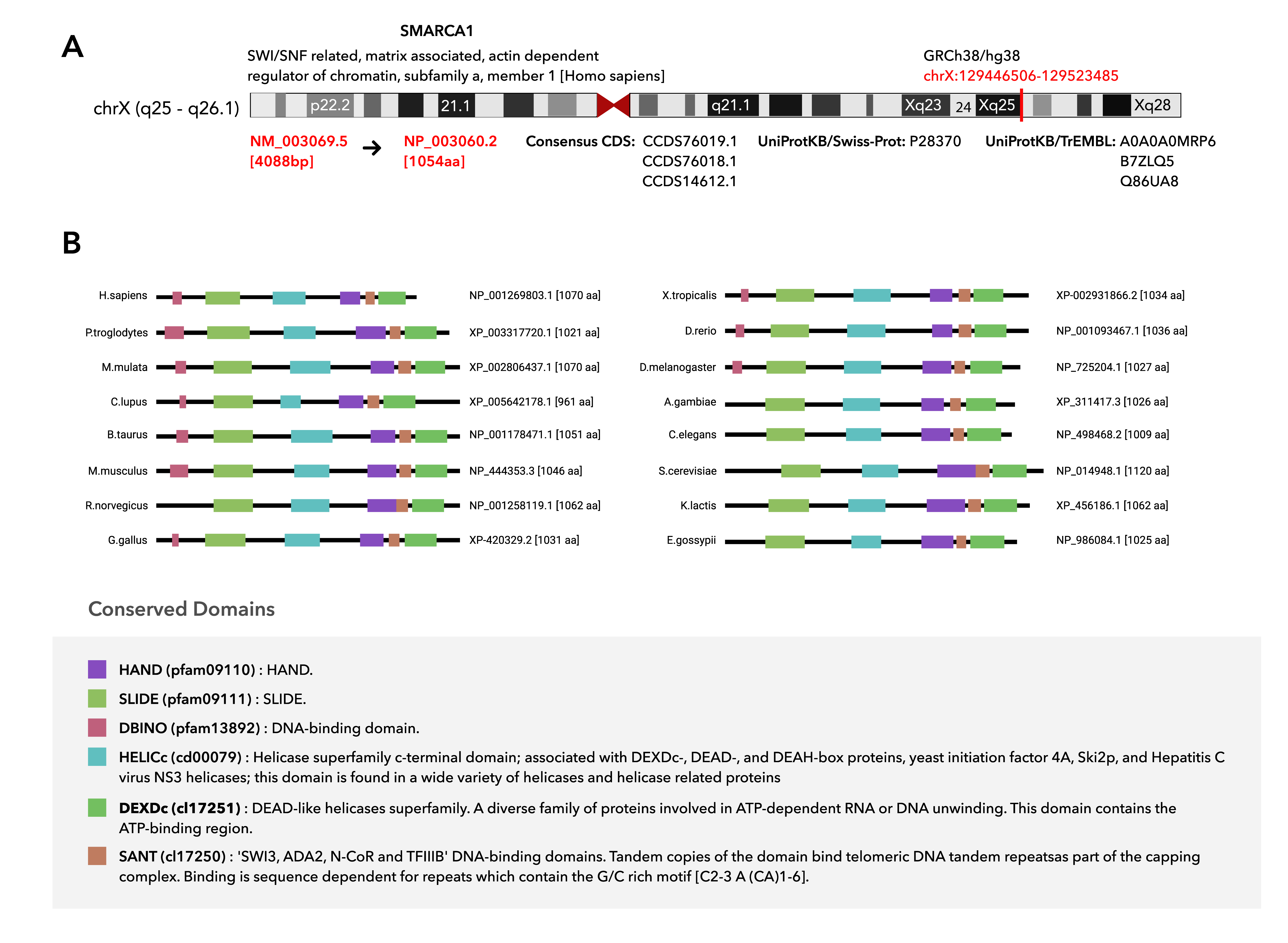

Supplement: S3 Fig — (A) Genomic location of human SMARCA1; (B) Conserved domains of SMARCA1 protein among diverse species. (TIF) [file pone.0274823.s003.tif]

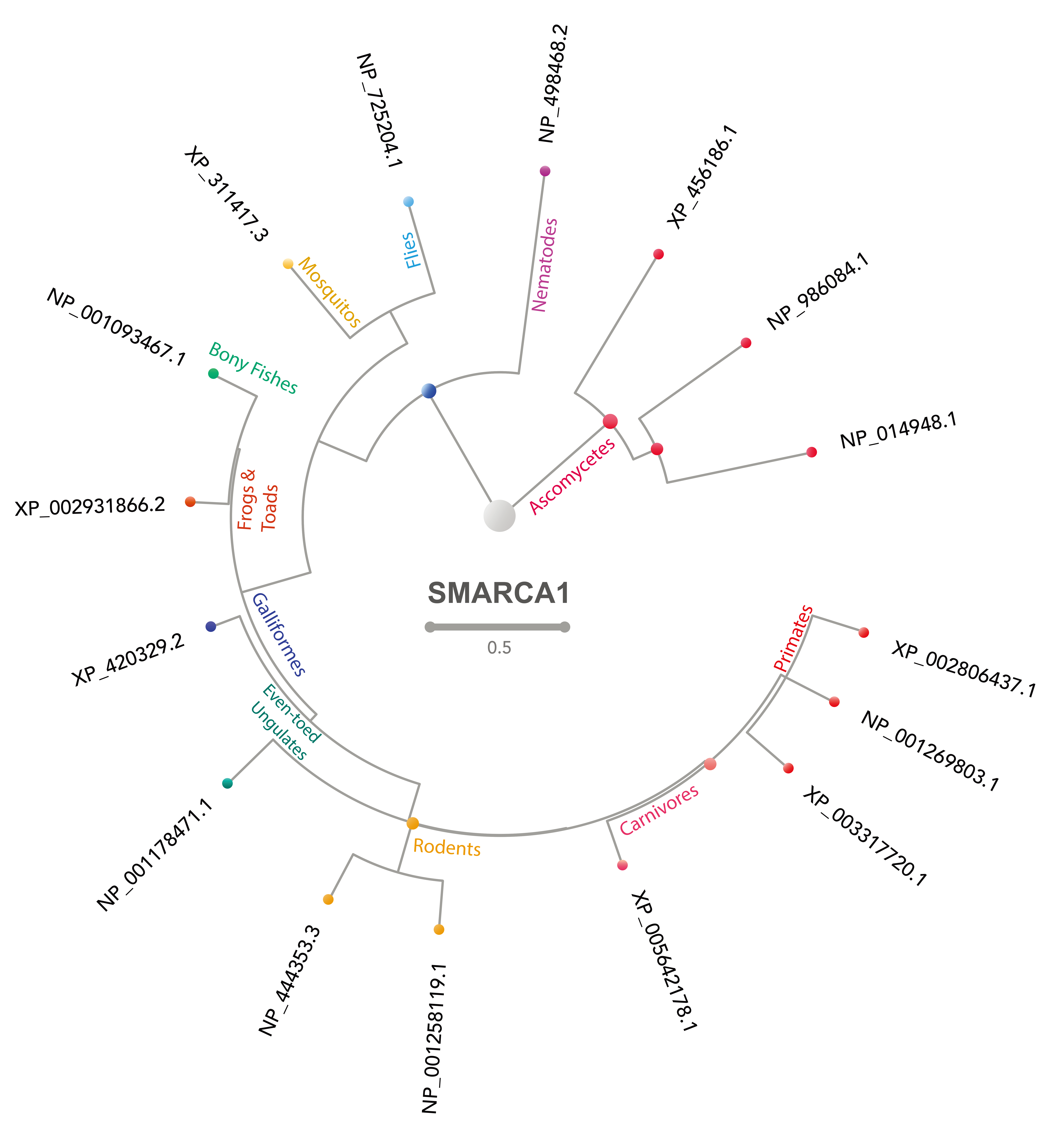

Supplement: S4 Fig — We obtained the phylogenetic tree of SMARCA1 in different species via COBALT of NCBI. COBALT, constraint-based multiple alignment tool. (TIF) [file pone.0274823.s004.tif]

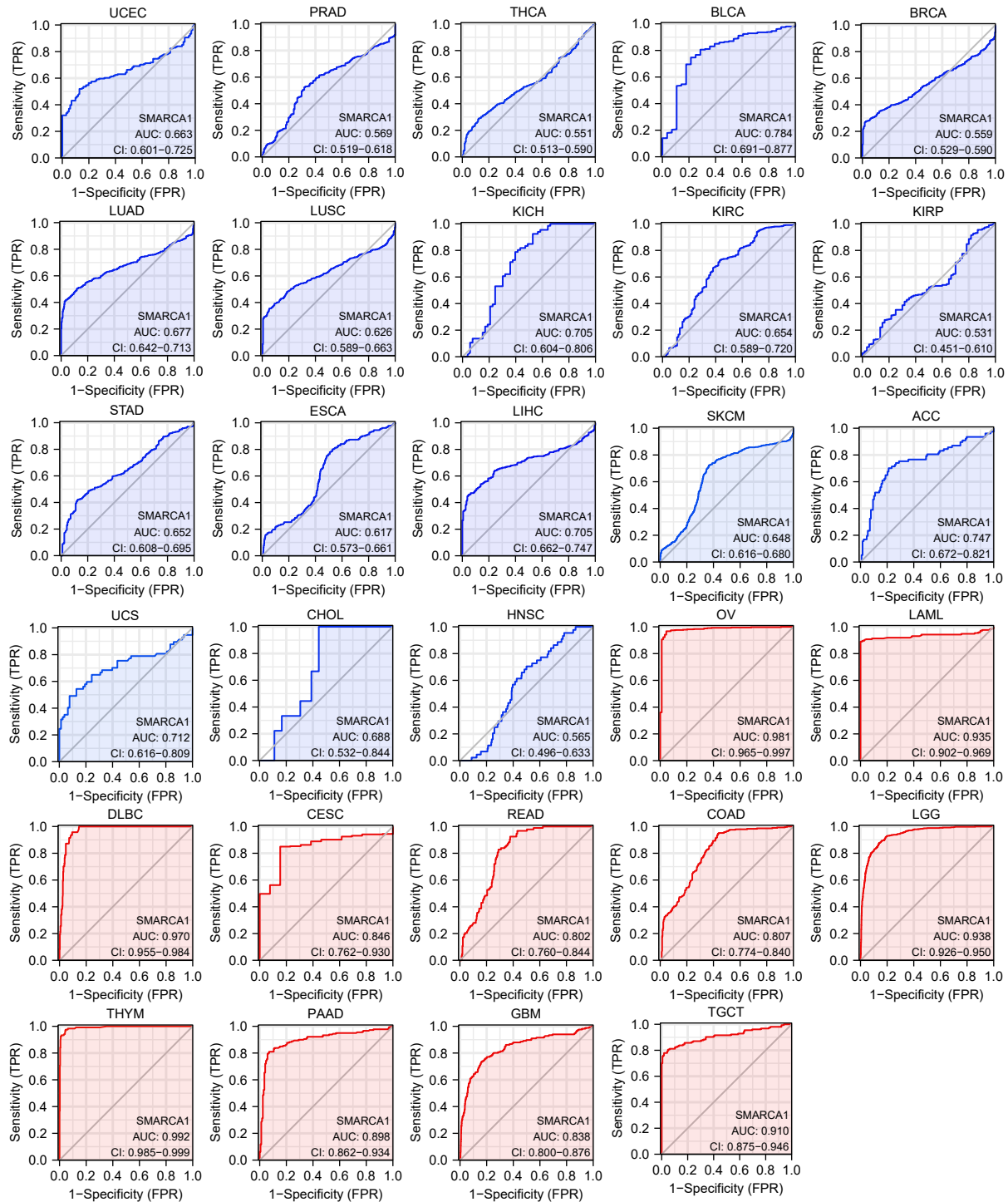

Supplement: S5 Fig — SMARCA1 had different diagnostic abilities in different tumors. AUC, area under curve. * P < 0.05, ** P < 0.01, *** P < 0.001. (PDF) [file pone.0274823.s005.pdf]
